# SLC38A10 Knockout Mice Display a Decreased Body Weight and an Increased Risk-Taking Behavior in the Open Field Test

**DOI:** 10.3389/fnbeh.2022.840987

**Published:** 2022-05-23

**Authors:** Frida A. Lindberg, Karin Nordenankar, Robert Fredriksson

**Affiliations:** Molecular Neuropharmacology, Department of Pharmaceutical Biosciences, Uppsala University, Uppsala, Sweden

**Keywords:** SLC38A10, SNAT10, risk-taking behavior, solute carrier, open field, behavior, elevated plus maze, phenotyping

## Abstract

The solute carrier 38 family (SLC38) is a family of 11 members. The most common substrate among these are alanine and glutamine, and members are present in a wide range of tissues with important functions for several biological processes, such as liver and brain function. Some of these transporters are better characterized than others and, in this paper, a behavioral characterization of SLC38A10^−/−^ mice was carried out. A battery of tests for general activity, emotionality, motor function, and spatial memory was used. Among these tests, the elevated plus maze, Y-maze, marble burying and challenging beam walk have not been tested on the SLC38A10^−/−^ mice previously, while the open field and the rotarod tests have been performed by the International Mouse Phenotyping Consortium (IMPC). Unlike the results from IMPC, the results from this study showed that SLC38A10^−/−^ mice spend less time in the wall zone in the open field test than WT mice, implying that SLC38A10-deficient mice have an increased explorative behavior, which suggests an important function of SLC38A10 in brain. The present study also confirmed IMPC's data regarding rotarod performance and weight, showing that SLC38A10^−/−^ mice do not have an affected motor coordination impairment and have a lower body weight than both SLC38A10^+/−^ and SLC38A10^+/+^ mice. These results imply that a complete deficiency of the SLC38A10 protein might affect body weight homeostasis, but the underlying mechanisms needs to be studied further.

## Introduction

The solute carriers (SLCs) are proteins belonging to the biggest group of transporters in vertebrates, and they have a wide range of substrates. Until today, 66 families have been discovered (Hediger and Gyimesi, 2004; Gyimesi and Hediger, 2021), but not every family is well-characterized. SLC38A10 is one of eleven members in the SLC38 family (SLC38A1-11). These amino acid transporters are referred to as Sodium-coupled Neutral Amino acid Transporters (SNATs), and their transport profiles are divided into two main systems: system A, which can be inhibited by 2-methylamino-isobutyric acid (MeAIB) and has quite a broad substrate profile, and system N with a narrower substrate profile than system A, but for which the transport also can be driven by H^+^ (Bröer, 2014). Hellsten et al. (2017) reported that SLC38A10 has a bidirectional transport of L-alanine, L-glutamate, L-glutamine and D-aspartate, and also an efflux of L-serine. SLC38A10 is also capable of transporting MeAIB, for which SNAT10 has been suggested to belong to system A, although the classification is complicated due to its similarities with system N (Hellsten et al., 2017).

The SNATs have different expression profiles and are associated with various functions *in vivo*, but are considered to be especially important for the glutamate-glutamine cycle in the brain (SNAT1, 2, 3, 5, and possibly 6, 7 and 8; Chaudhry et al., 1999; Boulland et al., 2002; Cubelos et al., 2005; Blot et al., 2009; Hägglund et al., 2011, 2015; Bröer, 2014; Gandasi et al., 2021) and for liver function, such as in the urea cycle and the glucose-alanine cycle in the interorgan cycle between liver and muscle (SNAT2, 3, 4 and 5; Chaudhry et al., 1999; Hatanaka et al., 2001; Varoqui and Erickson, 2002; Baird et al., 2004; Bröer, 2014). SNAT2, in the plasma membrane, and SNAT9, in the lysosomal membrane, have recently been recognized as sensors in the mTORC1 pathway and are important in the cellular response to amino acids (Evans et al., 2007; Pinilla et al., 2011; Jung et al., 2015; Rebsamen et al., 2015; Wang et al., 2015; Wyant et al., 2017).

So far, there have been a handful of studies focusing on the expression, substrate profile, and function of SLC38A10, but the results do not clearly point to its physiological function. In the rat, *Slc38a10* mRNA is expressed in most peripheral tissues and in the brain, with the highest expression in pituitary gland, eyes, and lungs (Sundberg et al., 2008). Another study in rats found *Slc38a10* mRNA expression all through the gastrointestinal tract (Cedernaes et al., 2011). In mice, SLC38A10 is abundantly expressed in the brain and found in both astrocytes and neurons. In addition, taken together with its substrate profile, it was suggested that SLC38A10 could be involved in excitatory and inhibitory neurotransmission (Hellsten et al., 2017). Interestingly, a recent study found SLC38A10 to be localized to intracellular organelles, mainly the endoplasmic reticulum and Golgi, and that an RNAi-knockdown of the transporter resulted in a reduction of protein synthesis. Hence they suggest SLC38A10 could function as a transceptor in a similar way as SNAT2 and SNAT9 (Tripathi et al., 2019). Furthermore, a study of a *Slc38a10* knockout mouse found that *Slc38a10* is linked to a decreased bone mineral content, and authors suggest that SLC38A10 might have a critical function in skeletal differentiation during development (Bassett et al., 2012). The International Mouse Phenotyping Consortium (IMPC) reported that mice deficient in SLC38A10 protein have a decreased body weight, along with altered levels of metabolic and hematopoietic markers in blood (Dickinson et al., 2016; Slc38a10, n.d.). The focuses so far have been to study the role of SLC38A10 in the periphery. However, there is a link between amino acid transport, brain function, and various psychiatric disorders (Flyckt et al., 2001; Smith et al., 2001; Tǎrlungeanu et al., 2016; Hu et al., 2020). For example, Guan et al. (2019) found SLC38A10 to be one of several genes associated with autism, and other members of the SLC38 family, e.g., SLC38A5 and SLC38A7, to be associated with schizophrenia. We therefore wanted to investigate if there is a link between SLC38A10 and brain function, and we did that by studying behavioral traits that can be associated with normal brain function and psychiatric disorders. By using a knockout-first allele (Testa et al., 2004; Skarnes et al., 2011) mouse model, we studied the behavior of mice deficient in the SLC38A10 protein in different behavioral paradigms targeting general activity, emotionality, motor function, and spatial memory. Due to SLC38A10 ubiquitous expression and earlier hypothesis of it's possible role in the glutamate-glutamine cycle in the brain (Sundberg et al., 2008; Hellsten et al., 2017), we wanted to further study the effects of SLC38A10 deficiency on risk-associated behaviors and cognitive function using the open filed test, elevated plus maze and the Y-maze. Due to the body weight difference previously reported, we also wanted to exclude motor impairments using a more sensitive motor function test than the rotarod by using the challenging beam walk test (Fleming et al., 2013). However, the rotarod was also used in order to replicate earlier finding by the IMPC (Dickinson et al., 2016; Slc38a10, n.d.) along with the crude grip strength test.

## Methods

### Animals

All experiments in the study using mice were approved by Uppsala Animal Ethical Committee (C69/15, C77/14 and 5.8.18-09820/2018) and followed Swedish Legislation on Animal Welfare. The animals were kept in an animal facility with a controlled environment, with humidity (45–65%), temperature (20–24°C), and ventilation system, and a 12-h light/dark cycle with lights on at 7 a.m. Animals were group housed in open cages, with two to five animals per Green Line cage (501–530 cm^2^) separated by sex, with wood-chip bedding, a cardboard house, and paper as enrichment. Breeding was carried out in type III cages (800–820 cm^2^). Mice had daily supervision and *ad libitum* access to food (pellets R3, Lantmännen, Sweden) and water.

Heterozygous (B6Dnk;B6N-Slc38a10^tm2a(EUCOMM)Wtsi^/H) and control mice were bought from the International Mouse Phenotyping Consortium (IMPC; Slc38a10, n.d.) and kept for in house breeding. The first heterozygous males from that breeding were bred with C57BL/6J females (Taconic M&B, Denmark). Further breeding was carried out from in house bred offspring.

In this paper, SLC38A10^−/−^ mice will be abbreviated as KO, SLC38A10^+/−^ as HET, and SLC38A10^+/+^ as WT.

### Genotyping

**Figure 1:**
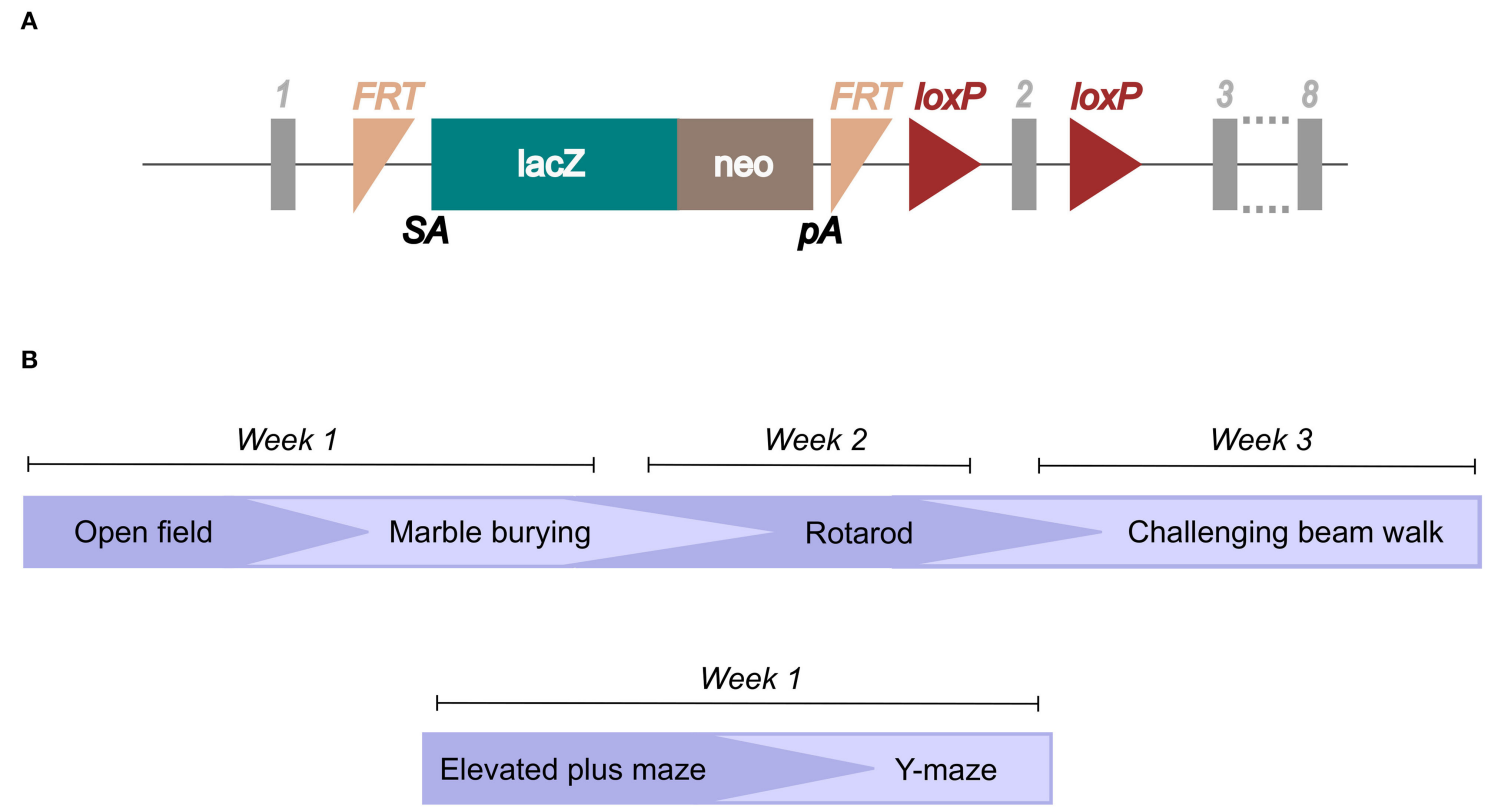
Illustration of the knockout construct and study layout. The knockout mouse was imported from the International Mouse Phenotyping Consortium (IMPC; www.mousephenotype.org/data/genes/MGI:1919305), **(A)** and the knockout-first construct is illustrated (Created using data from Slc38a10, n.d.). Genotyping primers were designed toward the LacZ domain and the wild-type allele. **(B)** The study layout for which mice went through the different behavioral tests in the order illustrated.

Ear biopsies were obtained after weaning (3–4 weeks of age) and incubated in 75 μl of 25 mM NaOH and 200 μM EDTA at 95°C for 40 min. After a cooling time of 5 min on ice, 75 μl of 40 mM Tris-HCl was added and the samples were then ready for PCR analysis. For the PCR, primers for the LacZ domain in the knockout construct (Figure 1A) and primers for the wild-type gene were designed.

### Embryo Size and Body Weight Measurement

Mated females were checked for a vaginal plug and, if present, that was determined as gestational day 0.5. At gestational day 15.5, the females was euthanized through cervical dislocation and embryos (16 WT, 25 KO) were dissected, photographed with a ruler, and decapitated. Measurements were made with ImageJ software. Weight data from the first 3 weeks of life were obtained from individual weighing of pups (males: 10 WT, 8 KO; females: 11 WT, 5 KO) at the same time every day, and was always carried out by the same person. Individual weighing of adult mice was carried out at the age of 10, 13, 20, and 25 weeks of age (males: 12 WT, 10 HET, 12 KO; females: 15 WT, 12 HET, 11 KO).

### Weighing of Food

Two mice of the same genotype were housed per Green Line cage, in which a pre-weighed amount of food was placed. Every 24 h, during the light period, the amount of food left was weighed and thereafter refilled and weighed again, for five consecutive days. The amount of food eaten was divided with total weight of the two mice, hence each cage is one statistical unit. Only WT and KO mice were included, and they were 8–9 weeks of age (9 WT cages: 3 cages with males; 6 cages with females; 7 KO cages: 3 cages with males; 4 cages with females).

### Behavioral Characterization

Both male and female mice were used, and behavior testing was carried out on WT, HET, and KO mice. Male mice were always tested prior to female mice, and cleaning was done between each mouse with 10% ethanol (unless otherwise stated). Mice were handled daily on the week before initiating the first behavioral test and always by the same person that performed the experiments (FL).

Handling protocol: The first 4 days of handling was carried out in the home room of the animals, with just a hand present in the cage for 5 min the first day. The same procedure was conducted during the second day, but without enrichment in the cage. During the third and fourth day of handling, the hand of the handler was placed in the cage for 2 min, without enrichment present in the cage, and each moused was lifted and put on the arm of the handler for 30 s. During the fourth day, each mouse was placed on the arm twice. During the fifth and final day of the handling protocol, the cages was placed in the experimental room for 30 min, and thereafter was each mouse handled in the same way as on the previous day.

Animals were habituated to the test room in their home cage (unless otherwise stated) 1 h prior to the start of each test. The elevated plus maze (EPM), Y-maze, and open field (OF) tests were recorded with the Ethovision system (version 11.0, Noldus Information Technology, Wageningen, the Netherlands). The same software was used for automatic tracking of the OF and EPM.

Animals were either going through the EPM and the Y-maze, or the OF, marble burying, rotarod, and the challenging beam walk, in respective order (Figure 1B). Mice used in the grip strength test were only used in that test. Littermate controls from heterozygous breeding were used for all tests, except for the grip strength test where KO and WT mice were derived from separate breedings. Mice were of age 7–9 weeks when they entered the first test, and it was at least 48 h between each test in case of repeated testing. All behavioral testing occurred during the light phase of the light/dark cycle.

#### Open Field

Open field is a common test used to study locomotor activity and emotionality by assessing thigmotaxis vs. center exploration (Gould et al., 2009). The arena can be either round or square, and for this study, a square 50 × 50 cm arena with high, black walls was used (illumination of 60–80 lux). Mice (males: 13 WT, 14 HET, 9 KO; females: 12 WT, 14 HET, 9 KO) were allowed to explore the arena for 60 min. Prior to analysis, the arena was divided into three different zones: central circle (ø18 cm), outer circle (ø37 cm; data not analyzed for this article), and wall zone (the rest of the arena). The 60-min period was divided into 5 min time bins when analyzed (referred to as *time-points*). Frequency of visits, cumulative duration (s), and mean velocity (cm/s) in the respective zone were scored, duration per visit was calculated, and total distance (cm) moved in the arena was automatically tracked. For mean velocity in the central circle only animals that entered the central circle at least once during each time-point were analyzed, resulting in a lower number of animals for this parameter (males: 5 WT, 9 HET, 6 KO; females: 5 WT, 10 HET, 4 KO). Frequencies of rearing and grooming were scored manually during the first 5 min of the test period (males: 13 WT, 15 HET, 14 KO; females: 15 WT, 15 HET, 10 KO). Animals that were excluded from the analysis of the whole 60-min period due to climbing on the walls of the OF arena were included in the first 5-min scoring of rearing and grooming, hence the difference in number of animals between these parameters. Fecal boli were counted for a subset of animals (males: 7 WT, 11 HET, 8 KO; females: 7 WT, 6 HET, 6 KO).

#### Elevated Plus Maze

The EPM offers a conflict between exploration of open and elevated arms vs. sheltered arms, where mice show a thigmotaxic behavior in the sheltered (closed) arms and a more risk-taking behavior on the open arms. This has led to that the EPM is traditionally seen as a test for anxiety-like behavior (Walf and Frye, 2009). In this study, the EPM had four arms in the shape of a plus sign, two of them had walls (*closed arms*, 40–50 lux) and the other two did not (*open arms*, 90 lux). Each arm was 35 cm long and 5 cm wide, creating a center of 5 × 5 cm in the middle of the maze (70 lux) which was not considered as either closed or open. The maze was elevated 50 cm above the floor. Animals explored the maze for 10 min, and frequency of visits and cumulative duration (s) in each arm were scored (males: 8 WT, 11 HET, 10 KO; females: 6 WT, 11 HET, 8 KO).

#### Marble Burying

In the marble burying test, mice are introduced to marbles and their burying behavior is measured by counting the number of marbles that are buried after a specified time. Burying is an innate behavior of mice, and the marble burying test has been argued to model changes in affect, although activity level is probably more accurate (Deacon, 2009). In this study, mice were single housed and habituated to the test room (90 lux) for 1 h in the cages in which they performed the test (males: 10 WT, 12 HET, 11 KO; females: 12 WT, 13 HET, 9 KO). The cages had a 5 cm layer of woodchip bedding and paper as enrichment during the habituation period. Prior to the test, the paper was removed and 18 glass marbles with a diameter of 15 mm were put in a regular pattern. Each mouse was in the cage for 15 min. Thereafter, the number of buried marbles were counted. Marbles buried to a minimum of 75% were considered buried. The marbles were cleaned with 10% ethanol before use in the next cage.

#### Y-Maze

The Y-maze is a test of spontaneous alternation and is used as a test of spatial working memory (Kraeuter et al., 2019). It is based on the innate exploratory drive of mice, particularly in exploring the arm it has not recently visited. The Y-maze arena used in this study had 50 cm long arms, and each arm was 5 cm wide (illumination of 50 lux in the arena). The mouse was put in the middle of the arena and explored for 10 min (males: 10 WT, 12 HET, 14 KO; females: 9 WT, 13 HET, 14 KO). When scoring, each arm was given a letter which gave rise to a sequence depending on how the animal moved in the maze. The sequence was analyzed by looking at the sequence in triplicates. Specifically, spontaneous alternation was defined as when the mouse entered 3 different arms in one triplicate. The spontaneous alternation score was obtained by dividing the alternations the mouse did with the number of possible alternations. An entry into an arm was defined as when the mouse had all paws in it. Total number of entries was used as a measure of activity.

#### Rotarod

Motor coordination and motor learning were tested by using the rotarod test (Roto-rod, IITC, California, USA). In this test, mice were put on a rotating rod, and the latency to fall was recorded (males: 9 WT, 13 HET, 11 KO; females: 12 WT, 13 HET, 9 KO). The first 3 days of the test consisted of a learning period with a constant speed of the rod at 24 rpm with a maximum time limit of 60 s. On the fourth day, the speed was accelerating from 4 to 40 rpm during a 2 min period, and the total length of the test was a maximum 3 min. Each animal was given four trials per day during the learning period and three trials on the test day, with at least 5 min rest between the sessions. Cleaning of the equipment was carried out between each animal with water and a mild detergent. Animals that fell off the rod after 3 s or less were excluded from the analysis.

#### Challenging Beam Walk

The challenging beam walk test is a test of sensorimotor function, which here was used as a complement to the rotarod test since it is useful to detect more subtle motor impairments than the rotarod (Fleming et al., 2013). The test consists of a 1 m long beam in four segments (25 cm/segment), where each segment is thinner than the previous one (35, 25, 15, and 8 mm wide, respectively). The beam was elevated ~40 cm, and at the thinner end, there was a goal house with woodchips from the home cage. Two days of training was required before the test day. During the first day, mice were habituated to the tapered beam. Mice were put in the goal house for 5 min and thereafter placed on the beam 20 cm away from the goal house. When the mouse learnt to enter the goal house, it was put further away and so on until it learnt to traverse the beam without hesitation. On day two, the mouse was put on the start position and traversed the beam two to four times. On day three, a mesh grid was put on the beam prior to the start of the test. Each mouse was given three trials (males: 7 WT, 11 HET, 7 KO; females: 10 WT, 11 HET, 7 KO), and each trial was recorded in slow motion (Panasonic HC-VX870, Panasonic, Osaka, Japan). Every step the animal took with its right foreleg was counted as the number of total steps, and each slip off the grid with each leg was counted as the number of errors.

#### Grip Strength

Animals were put on a cage lid that was turned upside-down to study the animals' grip strength. The test was terminated when the animal (i) fell (~30 cm) onto wood-chip bedding, (ii) climbed onto the upside of the cage lid, or (iii) the time limitation was reached. The cut off time was 3 min, and mice had three trials with at least 5 min rest in between. Animals in this test were 9–10 weeks of age and only WT and KO mice from separate breedings were used (males: 9 WT, 13 KO; females: 8 WT, 10 KO). The latency to fall (s) was noted, and the best trial was weight corrected and used in analysis. Trials terminated due to (ii), climbing onto the upside of the cage lid, were not included in the analysis.

### Statistical Analysis

Embryo size was analyzed with Mann-Whitney U-test and body weight data were analyzed with two-way ANOVA (repeated measures) and Bonferroni *post-hoc* tests (GraphPad Prism version 5.02, GraphPad software Inc., San Diego, California USA). A majority of the results from the behavioral tests were not normally distributed as shown by the Shapiro-Wilk normality test (GraphPad Prism, version 5.02), hence non-parametric statistics were used. The EPM, Y-maze, rotarod, challenging beam walk and grip strength tests were analyzed with the Kruskal–Wallis and Dunn's multiple comparisons test (GraphPad Prism, version 5.02). Longitudinal data sets (OF and food intake) were analyzed for main effects and interactions in R 4.0.4 (R Core Team, 2021) with the nparLD package (Noguchi et al., 2012), with genotype and sex as between-subject factors and time as within-subject factor. Significant effects and interactions were further examined in Statistica 13 (TIBCO Software Inc., Tulsa, OK, USA) with *post-hoc* Mann–Whitney *U*-test with continuity correction (between subject-dependent) and/or Wilcoxon's matched pairs test (within subject-dependent). Data are presented as means ± standard deviation (body weights) or medians with interquartile range (IQR; embryo size, food intake and all behavioral data), and the significance level used was 0.05 for all tests. Effect sizes were calculated for significant differences according to Fritz et al. (2012).

## Results

In the present study, embryo size, body weight, and food intake were studied in SLC38A10-deficient mice. Moreover, a thorough behavioral characterization using tests (Figure 1B) targeting general activity, emotionality, motor function and spatial memory was made in order to decipher the importance of SLC38A10. A series of either OF, marble burying, rotarod and challenging beam walk, or elevated plus maze and Y-maze was used in respective order for KO, HET and WT mice. Another batch of mice was used for test of grip strength in KO and WT mice. From heterozygous mating resulting in 10–12 litters, the inheritance of the genotypes was calculated to be close to mendelian ratio [32% WT, 43% HET and 25% KO (*n* = 69)].

### KO Mice Have a Reduced Body Weight Compared to WT and HET Mice

**Figure 2:**
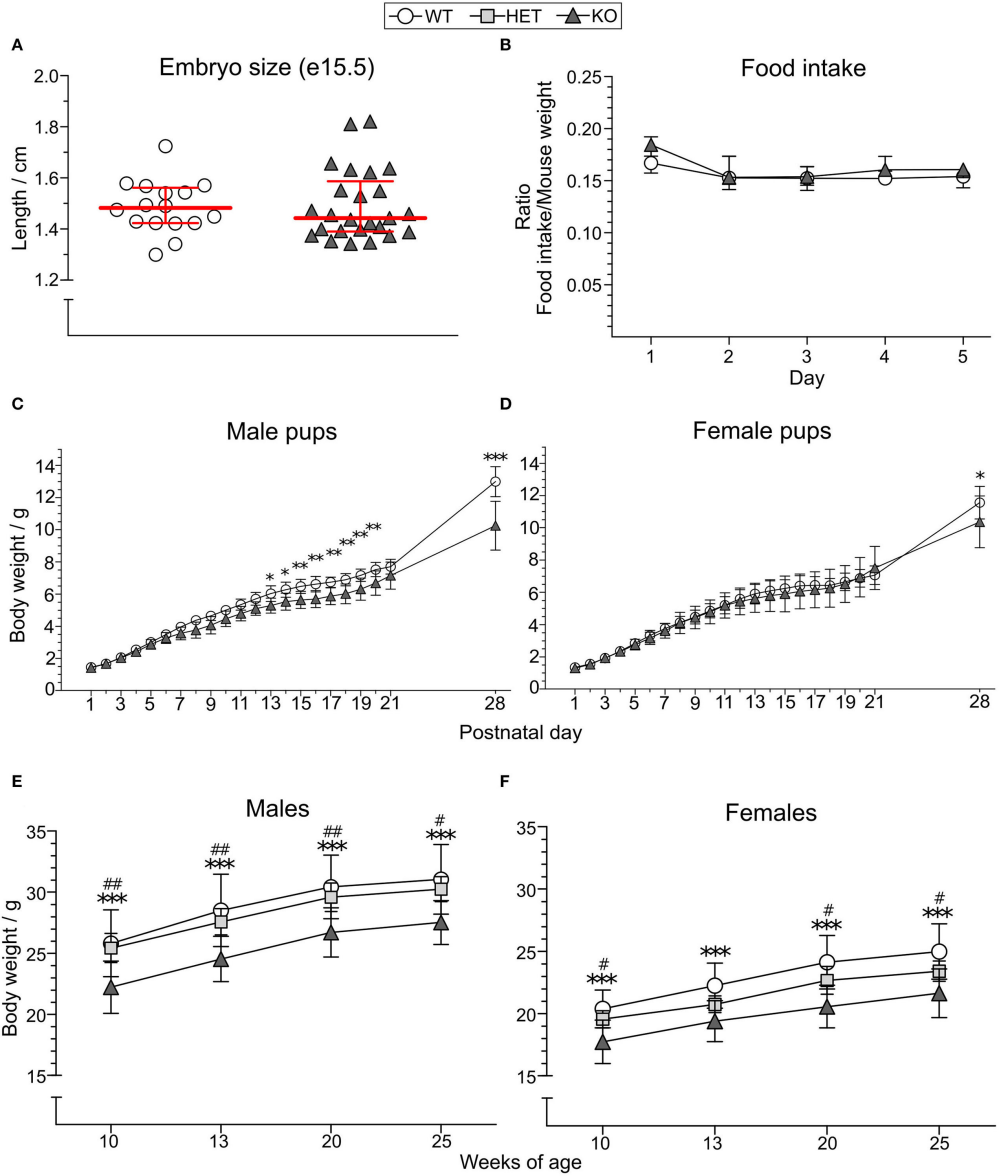
Effect of SLC38A10 knockout on food intake and body weight. **(A)** Embryos from SLC38A10^−/−^ mice (KO) (*n* = 25) and SLC38A10^+/+^ mice (WT) (*n* = 16) mice at gestational day 15.5 were dissected, photographed, and measured with ImageJ. No significant difference between the groups was observed. **(B)** Food pellets were weighed every 24 h for 5 consecutive days in mice housed two per cage (*n* = 9 WT, 7 KO cages) and a ratio of food eaten and mouse weight was calculated. Data in **(A,B)** are illustrated as medians with interquartile range. **(C)** Male (*n* = 10 WT, 8 KO) and **(D)** female (*n* = 11 WT, 5 KO) pups from KO and WT breedings were weighed (g) from postnatal day 1–28. **(E)** Male [*n* = 12 WT, 10 SLC38A10^+/−^ mice (HET), 12 KO] and **(F)** female (*n* = 15 WT, 12 HET, 11 KO) adult body weight (g) of KO, HET, and WT mice. Body weight data **(C–F)** are illustrated as means with standard deviation. Analyses were made with Mann–Whitney *U*-test for embryo size, food intake with the nparLD package in R, and a two-way ANOVA with Bonferroni *post-hoc* test for body weight measurements. Statistical differences between KO and WT mice are indicated with an asterisk sign (*), and between KO and HET mice with a hash sign (#), */#*p* < 0.05, **/##*p* < 0.01, ***p < 0.001.

Length of KO (median 1.443 cm, IQR 1.39–1.587, *n* = 25) and WT (median 1.483 cm, IQR 1.423–1.562, *n* = 16) embryos at gestational day 15.5 was used as an indicator of body size in the embryonal stage (Figure 2A) where no significant difference was observed. The food intake of KO (*n* = 7) and WT (*n* = 9) mice did not show any differences between the genotypes (main effect of genotype *p* > 0.5; main effect of time *p* < 0.01; main effect of sex *p* > 0.05; no interactions found) (Figure 2B, Supplementary Table 1). However, a significant body weight difference with lower body weight in KO than WT mice was found from postnatal day (PND) 13 in males (Figure 2C), with main effect of genotype at *p* < 0.01, main effect of time at *p* < 0.001, and interaction genotype:time at *p* < 0.001. In females, a body weight difference could be seen from PND 28 in females (Figure 2D), with no main effects of genotype at *p* > 0.05, main effect of time at *p* < 0.001, and interaction genotype:time *p* = 0.001. *Post-hoc* analyses showed that an increasing PND age discloses the genotype effect. The body weight of HET mice was not studied until adult age wherein a difference between KO and HET could be observed for most time points in both sexes [Figure 2E (main effect of genotype at *p* < 0.001; main effect of time at *p* < 0.001; no interactions found) and Figure 2F (main effect of genotype at *p* < 0.001; main effect of time at *p* < 0.001; interaction genotype:time *p* < 0.05)], while no differences were observed between HET and WT mice. *Post-hoc* analysis revealed that genotypes had the same effect for all time points except for the age of 13 weeks in HET female mice, explaining the interaction seen from the repeated measures ANOVA. Mean weights and standard deviations for each genotype and age are summarized in Supplementary Table 2.

### An Increased Risk-Taking Behavior in SLC38A10-Deficient Mice in the Open Field Test

**Figure 3:**
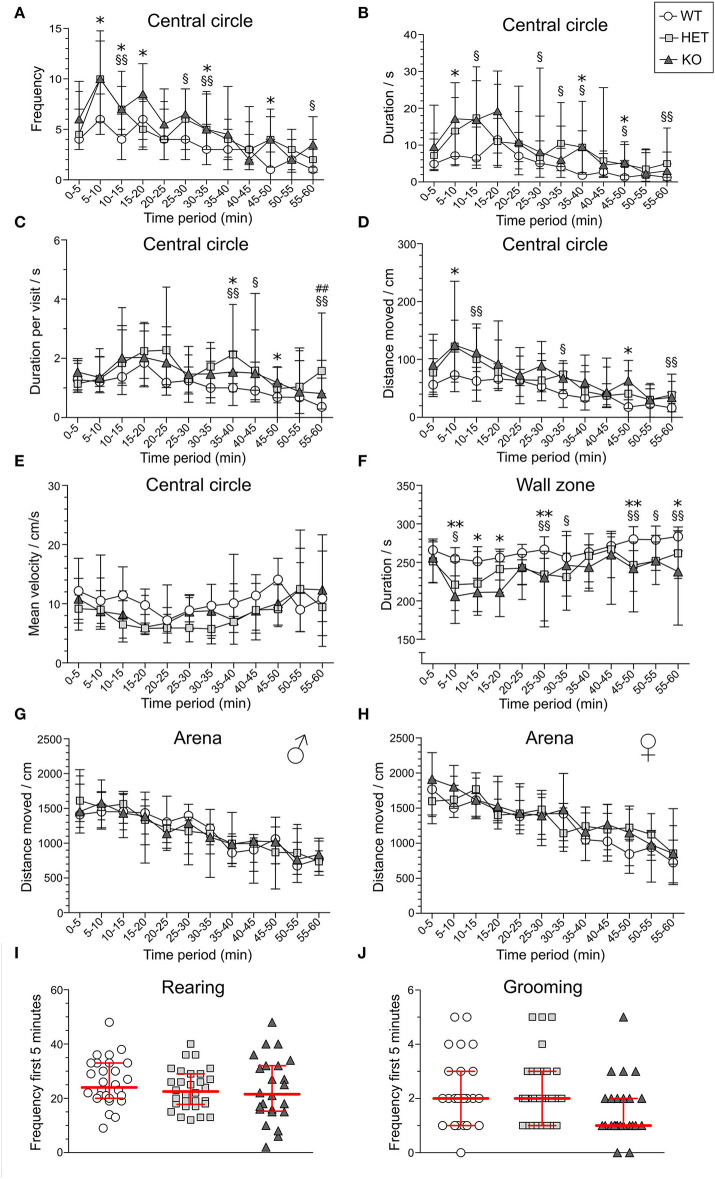
Behavior of SLC38A10-deficient mice in the open field (OF) test. Mice explored the arena for 60 min [*n* = 25 WT, 28 HET, 18 KO in **(A–D,F)**], and behavior in the central circle and wall zone was divided into 5 min time bins for **(A)** frequency, **(B)** duration (s), **(C)** duration per visit (s), **(D)** total distance (cm) moved and **(E)** mean velocity (cm/s) in the central circle (*n* = 10 WT, 19 HET, 10 KO), **(F)** duration (s) in wall zone, **(G,H)** total distance (cm) moved in the arena [*n* (males) = 13 WT, 14 HWT, 9 KO; *n* (females) = 12 WT, 14 HET, 9 KO], **(I)** number of rearings, and **(J)** number of grooming actions during the first 5 min of the test (*n* = 28 WT, 30 HET, 24 KO). In **(E)**, only animals that entered the central circle at least once per time-point were analyzed, which is why the number of animals is lower for this parameter. Data are illustrated as medians with interquartile range. Statistical differences between KO and WT mice are indicated with an asterisk sign (*), between HET and WT mice with a section sign (§) and between HET and KO mice with a hash sign (#), */§*p* < 0.05, **/§§##*p* < 0.01 (*post-hoc* Mann–Whitney *U*-test).

In the OF test, mice explored the arena for 60 min. The 60-min period was divided into 5-min time bins when analyzed (referred to as *time-points*), and medians and interquartile range of all groups are shown in Supplementary Table 3. Results are shown for male and female mice together for all parameters, except for total distance moved in the arena since it was influenced by sex (main effect of sex *p* < 0.01; Table 1). The main effect of time was significant for all parameters studied (*p* < 0.01; Table 1), while genotype had a main effect on frequency, duration, duration per visit, distance moved in central circle, and on duration in wall zone (*p* < 0.05; Table 1). Interactions were found for frequency and distance in the central circle (Sex:Genotype:Time, *p* < 0.05; Table 1). Statistics for each sex separately can be found for these parameters in Supplementary Table 3 and effect sizes can be seen in Supplementary Table 4. The most pronounced statistically significant genotype differences were seen between HET and WT mice, with KO mice following the same trend as HET mice. Relative to WT, SLC38A10-deficient mice showed increased amounts of visits to the central circle (at time-points 5–10, 10–15, 25–30, 30–35, 45–50, and 55-60 min; Figure 3A) along with an increased duration of time spent in the central circle (at 5–10, 10–15, 25–30, 30–35, 35–40, 45–50, and 55–60 min; Figure 3B, Supplementary Figure 1B), but not an increased duration per visit during the first half of the test (Figure 3C), indicating that SLC38A10 KO mice visited the central circle more often but did not spend more time there when visiting. For the second half of the test, SLC38A10-deficient mice had an increased duration per visit in the central circle than WT mice (at time-points 35–40, 40–45, 45–50, and 55–60 min; Figure 3C). The distance moved within the central circle was increased in HET and KO mice as a general trend, although it was only statistically significant for a few of these time points (at 5–10, 10–15, 30–35, 45–50, and 55-60 min; Figure 3D). Time spent in the wall zone was clearly affected by genotype, where both KO and HET mice spent less time than WT (Figure 3F, Supplementary Figure 1C). Effect sizes were calculated for statistically significant differences (Table 2), which indicated that the differences seen were either small or intermediate in size. Mean velocity in the central circle and total distance moved in the whole arena was not influenced by genotype (Figures 3E,G,H), although visual inspection of the graph indicates a trend toward a reduced velocity of SLC38A10-deficient mice in the central circle (Figure 3E). The frequency of rearing and grooming during the first 5 min of the OF test revealed no significant effect of genotype (Figures 3I,J). Fecal boli were counted after 60 min in the arena but showed no differences between the genotypes (Supplementary Figure 1A). These data together suggests that SLC38A10-deficient mice were more risk-taking than their WT littermates.

**Table 1:** Statistical main effects and interactions of genotype, time and sex in the open field (OF) test.

**Parameter**	**Main effect**	**Interaction**
Central circle, velocity	Time**	–
Central circle, frequency	Genotype**, Time***	Sex:Genotype:Time*
Central circle, duration	Genotype**, Time***	–
Central circle, distance	Genotype*, Time***	Sex:Genotype:Time**
Central circle, duration per visit	Genotype*, Time***	–
Wall zone, duration	Genotype**, Time***	–
Total distance	Sex**, Time***	–

*p < 0.05, **p < 0.01, and ***p < 0.001 in the R package nparLD.

**Table 2:** Calculated effect sizes for significant results in the OF test, ranging from small (0.1–0.3) to intermediate (0.3–0.5) effects (with no regard to minus sign; *n* = 25 WT, 28 HET, and 18 KO).

**Parameter**	**Time-point (min)**	**Comparison**	**Effect size**
Wall zone	5–10	WT vs. HET	0.28
duration	25–30	WT vs. HET	0.39
	30–35	WT vs. HET	0.28
	45–50	WT vs. HET	0.38
	50–55	WT vs. HET	0.34
	55–30	WT vs. HET	0.37
	5–10	WT vs. KO	0.42
	10–15	WT vs. KO	0.32
	15–20	WT vs. KO	0.35
	25–30	WT vs. KO	0.41
	45–50	WT vs. KO	0.45
	55–60	WT vs. KO	0.36
Central circle	35–40	WT vs. HET	−0.37
D/F	40–45	WT vs. HET	−0.27
	55–60	WT vs. HET	−0.37
	35–40	WT vs. KO	−0.31
	45–50	WT vs. KO	−0.30
	55–60	HET vs. KO	0.38
Central circle	10–15	WT vs. HET	−0.41
distance	30–35	WT vs. HET	−0.31
	55–60	WT vs. HET	−0.38
	5–10	WT vs. KO	−0.37
	45–50	WT vs. KO	−0.37
Central circle	10–15	WT vs. HET	−0.32
duration	25–30	WT vs. HET	−0.28
	30–35	WT vs. HET	−0.33
	35–40	WT vs. HET	−0.33
	45–50	WT vs. HET	−0.29
	55–60	WT vs. HET	−0.40
	5–10	WT vs. KO	−0.33
	35–40	WT vs. KO	−0.35
	45–50	WT vs. KO	−0.35
Central circle	10–15	WT vs. HET	−0.36
frequency	25–30	WT vs. HET	−0.32
	30–35	WT vs. HET	−0.39
	55–60	WT vs. HET	−0.33
	5–10	WT vs. KO	−0.35
	10–15	WT vs. KO	−0.31
	15–20	WT vs. KO	−0.30
	30–35	WT vs. KO	−0.30
	45–50	WT vs. KO	−0.34

### The Elevated Plus Maze

**Figure 4:**
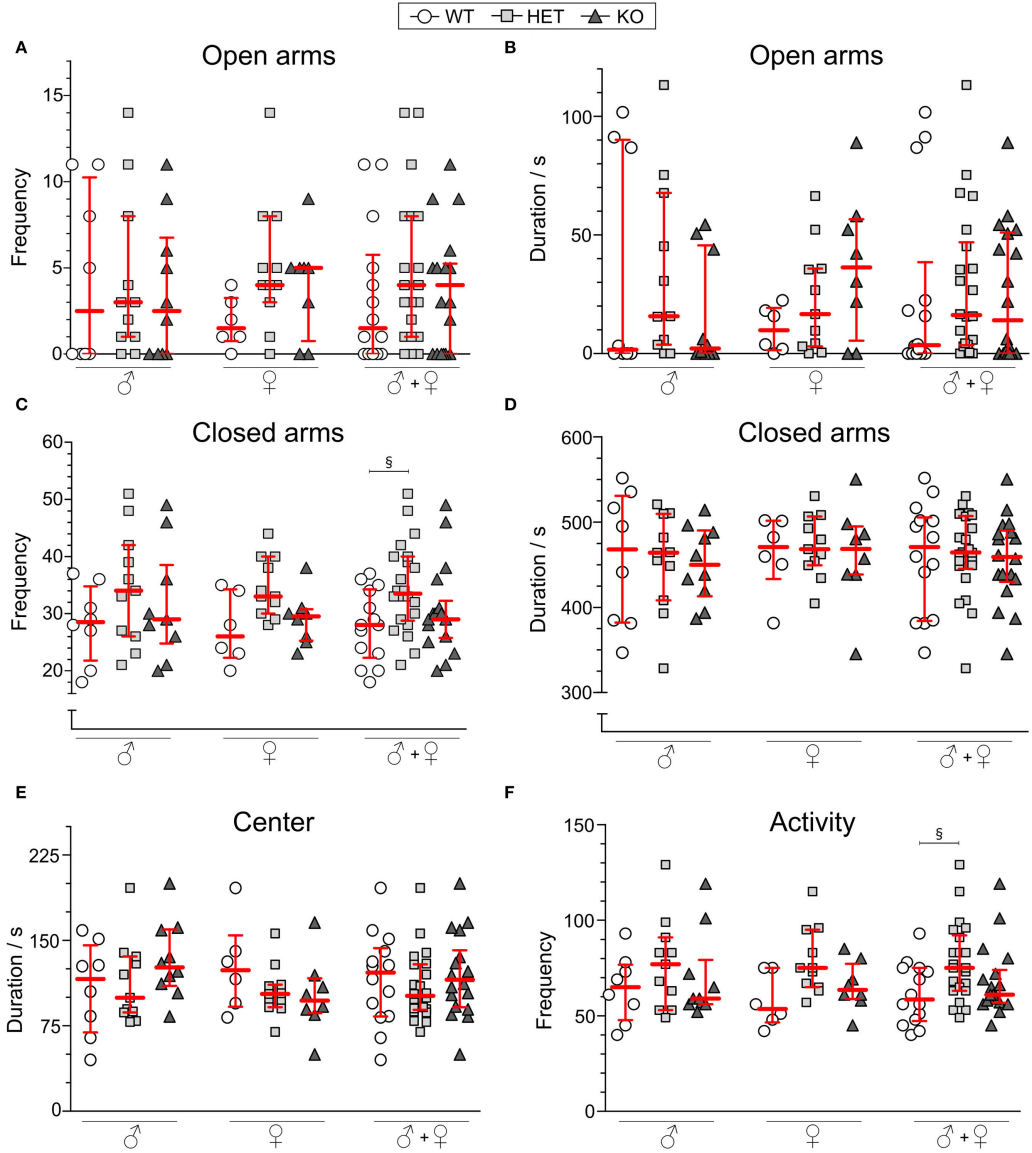
Behavior in the elevated plus maze of SLC38A10-deficient mice. Mice explored the elevated plus maze (EPM) for 10 min [*n* (males) = 8 WT, 11 HET, 10 KO; *n* (females) = 6 WT, 11 HET, 8 KO], and **(A)** frequency and **(B)** duration in *open arms* and **(C)** frequency and **(D)** duration in *closed arms* are plotted for each sex separately and together. **(E)** Duration in center of the EPM is plotted as well for sexes separately and together. As a measure of activity **(F)**, total frequencies in the arena were analyzed (sum of frequencies in center, open and closed arms). Data are shown as medians with interquartile range. Statistical differences between WT and HET mice are illustrated with the section sign (§). Analyses were made with the Kruskal–Wallis and Dunn's multiple comparisons test, §*p* < 0.05.

For 10 min, mice explored the EPM and frequency and duration in open and closed arms were analyzed along with center time. As shown in Figures 4A,B, the genotype did not affect the frequency of visits or the cumulative duration of time spent in open arms. Neither did the frequency of visits nor cumulative duration in the closed arms (Figures 4C,D) for each sex separately, but with sex collapsed HET mice displaying more visits to the closed arms than WT mice (Figure 4C). Duration in center was not affected by genotype (Figure 4E). The sum of visits to the center, open, and closed arms (Figure 4F) was analyzed as a measure of general activity, which, for the group with sex collapsed, showed an increased activity in HET mice compared to WT mice. Frequency in the center were found to be increased in HET mice with sex collapsed (Supplementary Table 5).

### Motor Activity, Grip Strength, Y-Maze and Marble Burying Behavior

**Figure 5:**
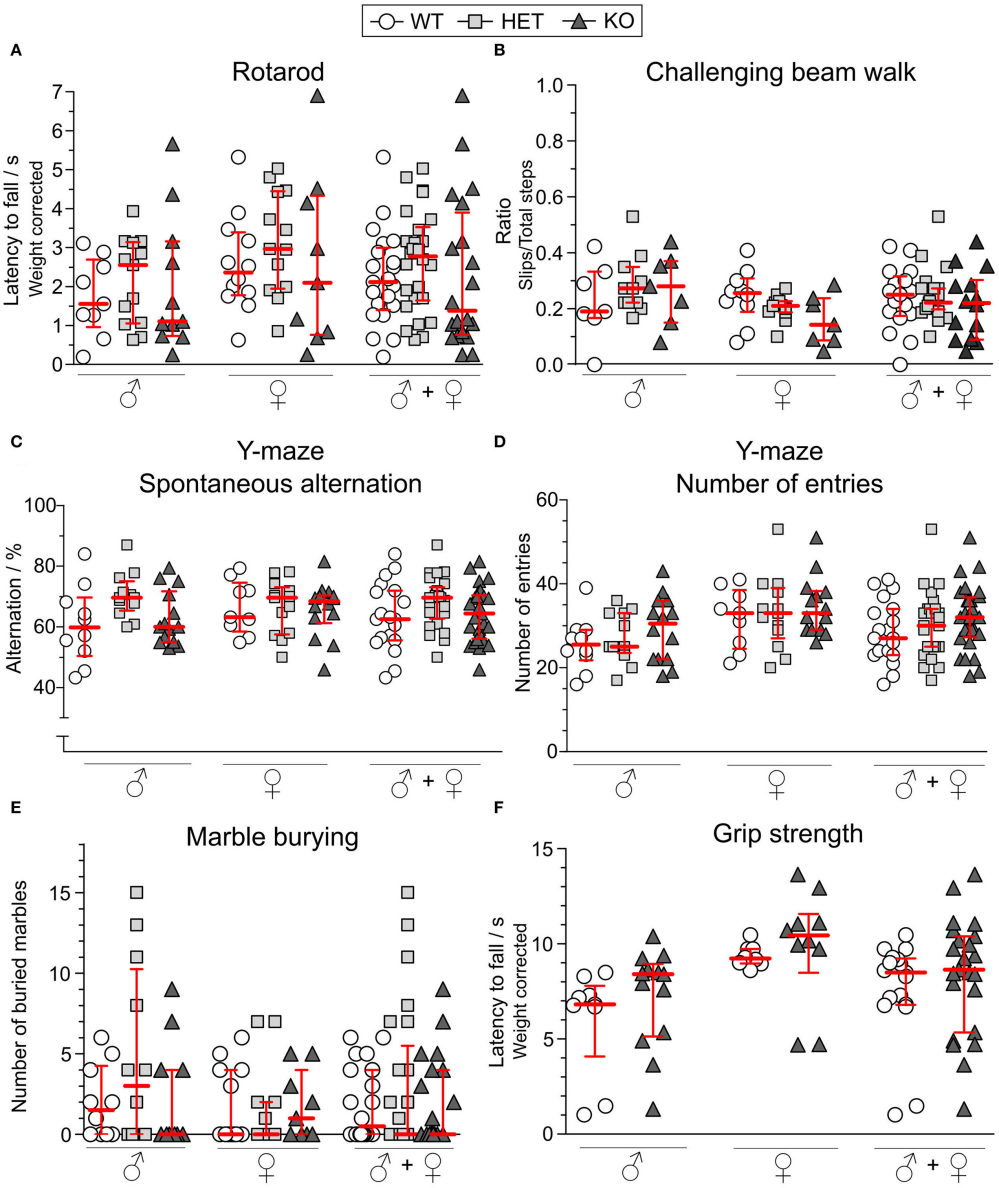
Motor function, Y-maze behavior and marble burying are unaffected in SLC38A10-deficient mice. For studying the motor function, **(A)** the rotarod [*n* (males) = 9 WT, 13 HET, 11 KO; *n* (females) = 12 WT, 13 HET, 9 KO] and **(B)** challenging beam walk [*n* (males) = 7 WT, 11 HET, 7 KO; *n* (females) = 10 WT, 11 HET, 7 KO] tests were used. Prior to the test day, animals had 3 (rotarod) respecitve 2 (challenging beam walk) days of training. On the test day, animals had 3 trials, and the best trial was used in analysis. For the rotarod data, results were weight corrected. For the challenging beam walk, the slip ratio was calculated from slips with all paws and from total steps with the right forepaw of the mouse. In the Y-maze [*n* (males) = 10 WT, 12 HET, 14 KO; *n* (females) = 9 WT, 13 HET, 14 KO], **(C)** spontaneous alternation and **(D)** number of entries were observed during 10 min of exploring the maze. **(E)** In the marble burying test [*n* (males) = 10 WT, 12 HET, 11 KO; *n* (females) = 12 WT, 13 HET, 9 KO], the mice were introduced to 18 marbles, and marbles buried to at least 75% were counted after 15 min. **(F)** Weight-corrected results from the grip strength test [*n* (males) = 9 WT, 13 KO; *n* (females) = 8 WT, 10 KO], where animals hang upside-down on a grid for a maximum of 3 min. Results from these tests are shown for each sex separately and together and are presented as medians with interquartile range. The genotype did not affect the mice's ability to perform in either of the tests. Analyses were made with the Kruskal–Wallis and Dunn's multiple comparisons test.

As a motor coordination test, the rotarod was used. After three consecutive days of training, mice had three trials on the test day from which the best trial was analyzed. As seen in Figure 5A, the genotype did not affect the performance in this test for either males or females. In the challenging beam walk test, a tapered beam with a mesh grid was used to study the mice's ability to coordinate their movement in a more challenging manner. The best trial out of three on the test day was analyzed for both sexes separately and together, but the KO or HET mice did not differ in their performance compared to WT mice (Figure 5B).

Spontaneous alternation is an innate behavior of mice, and is a trait that is used in the Y-maze to study spatial memory. The mice explored the Y-maze arena for 10 min and each visit to each arm was scored. The alternation score (Figure 5C) was not affected by either sex or genotype. The total number of entries into the arms was used as an activity measure but did not differ between the experimental groups (Figure 5D).

Repetitive behaviors were studied in the marble burying test in which mice were introduced to 18 glass marbles on woodchip bedding. For 15 min, mice investigated the marbles and marbles buried to a minimum of 75% were counted as buried ones. As observed in Figure 5E, either sex or genotype affected the number of buried marbles.

To test the grip strength, mice were placed on a grid and turned upside-down for a maximum of 3 min. No HET mice were included. No differences were found between KO and WT mice in male or female mice (Figure 5F).

Descriptive statistics for each of these tests can be found in Supplementary Table 6.

## Discussion

In the present study, the importance of SLC38A10 for embryo size, body weight, food intake and behavior in a range of different tests of general activity, emotionality, motor function and spatial memory were measured. The main findings were that KO mice had a lower body weight than both WT and HET mice, while food intake and embryo size were unaffected. SLC38A10-deficient mice also showed an increased risk-taking behavior in the OF test, but no other behavioral differences were found.

### SLC38A10 Knockout Mice Have a Decreased Body Weight

The SLC38A10 KO mouse showed a lower body weight than both the WT and the HET mice (Figures 2E,F), which agrees with previous data by IMPC (Dickinson et al., 2016; Slc38a10, n.d.). Since the focus of this study was to characterize the effects of SLC38A10 knockout on behavior, we did not investigate the lower body weight further. However, previous data (Bassett et al., 2012; Dickinson et al., 2016; Slc38a10, n.d.) indicate that the lower body weight is due to a decreased lean body mass. The normal weight of HET mice may indicate that one functional *Slc38a10* allele is enough to maintain a normal body weight and, possibly, bone mineralization. Herein, the differences in body weight between KO and WT mice were first seen at postnatal day (PND) 13 in males, and not until PND 28 in females (Figures 2C,D). The delay in females is probably due to the lower body weight in females compared to males, which makes a larger number of animals needed to detect differences. Since just five animals were used in the female KO group, it is possible that this body weight difference could have been detected earlier if more animals were used. When looking at when SLC38A10 is starting to be expressed during development, there are annotations of it to be expressed as early as at Theiler stage 12 (e7.5-8.75) (Smith et al., 2019). The body weight difference was not observed until 24–39 days later (depending on sex) based on our data, which is probably due to the low number of animals per group (as previously discussed). In order to detect size and/or weight differences earlier than we did, a much bigger *n* would probably be needed. On another note, the breeding strategy for collection of pup weight data was with homozygous breeding couples, and we cannot exclude the possible effect of the low body weight in the female as a confounding factor in these results. Previous studies have suggested *Slc38a10* to be important for cell volume regulation during development (Bassett et al., 2012).

Disruptions in function of other members of the SLC38 family, such as SNAT3 in mouse (Chan et al., 2016) and SLC38A11 ortholog in *D. melanogaster* (Aggarwal et al., 2020), also affect body weight, indicating the importance of SLC38 transporters.

### An Increased Risk-Taking Behavior in the Open Field Test, but Not in the Elevated Plus Maze

In the OF, both KO and HET mice spent less time in the wall zone, accompanied by more visits, longer duration per visit, and longer distance moved in the central circle in comparison to WT mice (Figure 3). The percentage of cumulative time spent in the central circle during the whole test period (60 min) was 2.1, 4.2, and 4.1%, and for time spent in wall zone, 86, 76.3, and 72.7% for WT, HET, and KO mice, respectively (Supplementary Figures 1B,C). Thus, the results suggest a more explorative and risk-taking behavior relative to WT. This is further supported by the lower, but non-significant, mean velocity in the central circle in HET and KO mice. Since the total distance did not differ between the genotypes, it is implied that the activity itself was not the cause of the increased visits to the center. Previous results have shown that animals with increased risk-taking behavior display a similar pattern (Momeni et al., 2014). Thus, the results imply that SLC38A10 could be involved in processes affecting risk-taking and explorative behavior. Fecal boli are sometimes used as a measure of emotionality (Hall, 1934), with a positive correlation between defecation and emotionality, but no differences were observed in this study (Supplementary Figure 1A), supporting the interpretations of the above discussed data. However, when calculating effect sizes of the significant results from the OF test (Table 2), the effects seen are small to intermediate, indicating that the significant results seen might not cause any major behavioral shift in the HET and KO mice. However, the increased risk-taking behavior observed in the OF was not reflected in the EPM. Either frequency of visits or the cumulative duration spent on the open arms in the EPM differed between the genotypes, for either of the sexes separately or together (Figures 4A,B,D). However, a genotype effect was seen on visits to the closed arms in HET mice in the analysis with sex collapsed (Figure 4C). The same effect was seen for the activity parameter (sum of all frequencies), indicating a slightly increased activity in HET mice. In males, the large individual variation in open arm behavior of the WT group impacted on the analysis, which makes the interpretation hard. If visually inspecting the graphs, one can speculate that there might be a tendency to an increase of time spent on the open arms in SLC38A10-deficient female mice, but it needs further studies to establish if there is an effect of genotype or not in this test.

To draw any conclusions from this data is troublesome due to the lack of a clear phenotype. The data indicates that there is a possibility of a more explorative/risk-taking behavior in SLC38A10 mice, but data from the OF test and the EPM do not point in the same direction. However, it is possible that the OF and EPM do not measure the same aspects of emotionality, which have previously been shown by the lack of correlations between them (O'Leary et al., 2013). It has also been previously suggested that locomotor activity can influence anxiety-related behavior, where results regarding emotionality need to be interpreted with caution due to its high correlation to activity (Dawson and Tricklebank, 1995; Milner and Crabbe, 2008). In our data, we do not see a different activity pattern in SLC38A10-deficient mice compared to their WT littermates in the OF test (see Figures 3G,H), suggesting that the differences between the genotypes seen is rather an effect on emotionality than locomotor activity. HET mice in the EPM were more active than both WT and KO mice (Figure 4F), but no aspects of risk-taking/thigmotaxic behavior could be observed. In the OF test though, females were a bit more active than males (Figures 3G,H), but this was not affected by genotype. Sex differences in locomotion have been reported before (Broida and Svare, 1984).

Amino acid transporters of various kinds are known to be implicated in neurodegenerative and psychiatric diseases (Smith et al., 2001; Tǎrlungeanu et al., 2016; Guan et al., 2019; Hu et al., 2020), which makes gene characterization research like the study conducted here needed. The altered behavior in the OF test could be due to the changed amino acid homeostasis that most likely exists in mice deficient in SLC38A10. If one considers the hypothesis by Tripathi et al. (2019) that SLC38A10 might function as a transceptor within the cell, likely as a glutamine sensor, one could hypothesize that flawed glutamine sensing would lead to a disturbance in glutamine/glutamate/GABA levels. Due to the importance of these systems, it is likely that there are compensatory mechanisms in place to correct flaws within them, or that additional systems for sensing also exists, and hence give rise to a milder phenotype as the one we see in this study (Tautz, 1992; El-Brolosy and Stainier, 2017). However, the SLC38A10 protein has been associated with Alzheimer's disease, and a single nucleotide polymorphism (SNP) and differences in methylation of the gene have been, respectively, found in patients with frontotemporal dementia and schizophrenia (Ferrari et al., 2015; Montano et al., 2016; Hashimoto et al., 2019), suggesting a possible role of the SLC38A10 protein in cognitive function. Based on the results from this study and the studies earlier mentioned associating SLC38A10 to cognitive function, further studies regarding these behaviors would be of interest, e.g., a memory test such as the Barnes maze. A multivariate test approach, like the multivariate concentric square field™ test (Meyerson et al., 2006), could be an interesting alternative to further study the behavioral profile of the SLC38A10 KO mouse.

### General Discussion

What is interesting to note is that HET mice are more similar to WT mice when comparing body weights (Figure 2). However, when looking at the behavior in the OF test, HET mice are behaving more like the KO mice. These data indicate that there might be enough of the SLC38A10 protein in HET mice to maintain a normal body weight, but not enough to not affect behavior in an OF test. One explanation for this could be that maintaining a normal body weight is more acute for the health of the animal, while the mild behavioral phenotype seen in this study is not as profound to affect the survival of the animal. In the long run, an increased risk-taking behavior could, of course, affect an individual's survival in the wild.

Regarding rearing, according to Sturman et al. (2018), unsupported and supported rears are to be seen as two distinct behavioral variables, and both should always be scored individually. In this study, the rearings scored were total rears, both unsupported and supported together. Hence no interpretation of the type of rearing occurring in each experimental group could be done. Rearing and stretch-attend-postures (SAPs) are behaviors that has been proven being more correlated between tests than the individual tests themselves (O'Leary et al., 2013), which was not scored for all tests (rearing) or at all (SAPs). It is also possible that mice who went through several behavioral tests might have been influenced by the previous test, which in turn could have influenced test results (McIlwain et al., 2001). However, by using the current study layout, we could decrease the number of animals needed. The inter-test interval of at least 48 h has before been proven to work just as well as one to two weeks in a specific behavior test battery (Paylor et al., 2006), which support the inter-test time in this study to be sufficient.

## Conclusion

Amino acid transport and amino acid homeostasis are without a doubt important for a healthy, functional organism. Even though we did not find any severe phenotype of SLC38A10-deficient mice in this study, we see an altered behavior in the OF test where KO and HET mice act less thigmotaxic, which suggests an increased risk-taking behavior. However, this was not a behavior that we could see in the EPM, which makes the interpretation less clear. The KO mice had a lower body weight than both WT and HET mice, which previous studies (Bassett et al., 2012; Dickinson et al., 2016; Slc38a10, n.d.) have reported is due to a decreased lean body mass. It would therefore be interesting to look further into the metabolism of these animals, which could give us a more complete understanding of SLC38A10 function, and to also conduct further tests of cognitive function and risk-taking behaviors.

## Data Availability Statement

The original contributions presented in the study are included in the article/Supplementary Material, further inquiries can be directed to the corresponding author/s.

## Ethics Statement

The animal study was reviewed and approved by Uppsala Animal Ethical Committee and followed Swedish Legislation on Animal Welfare.

## Author Contributions

FL planned the project, performed the experiments and analyses, and drafted the manuscript. KN planned the project, aided in videotaping the challenging beam walk test, read and approved the manuscript. RF planned the project, funded the study, and wrote parts of the manuscript. All authors contributed to the article and approved the submitted version.

## Funding

This study was supported by the Swedish Brain Foundation (Grant No. FO2018-01), the Novo Nordisk Foundation (Grant No. 16184), Åhlens Foundation, Gunvor and Josef Anér Foundation, and the Swedish Research Council (Grant No. 2016-01972).

## Conflict of Interest

The authors declare that the research was conducted in the absence of any commercial or financial relationships that could be construed as a potential conflict of interest.

## Publisher's Note

All claims expressed in this article are solely those of the authors and do not necessarily represent those of their affiliated organizations, or those of the publisher, the editors and the reviewers. Any product that may be evaluated in this article, or claim that may be made by its manufacturer, is not guaranteed or endorsed by the publisher.
