# Supplementary material for: SLC38A10 Knockout Mice Display a Decreased Body Weight and an Increased Risk-Taking Behavior in the Open Field Test
Source: Front Behav Neurosci. 2022 May 23;16:840987. doi: 10.3389/fnbeh.2022.840987 (PMC9169716; doi:10.3389/fnbeh.2022.840987)
Supplement: Supplementary file 1 [file Data_Sheet_1.pdf]

Supplementary Material

Table S1. Medians and the interquartile range for each day of the food intake measurement. The amount of food eaten was divided with total weight of the two mice in the cage. A main effect of time was found to be statistically significant, but no effect on genotype was observed (nparLD package, R 4.0.4).

Test	Time-point	Sex	WT Median	WT Quartiles	KO Median	KO Quartiles
Food intake	Day 1	Males	0,1606	0,1542-0,1671	0,1752	0,1566-0,1945
		Females	0,1810	0,1605-0,2075	0,1847	0,1764-0,1872
		(Males + Females)	0,1671	0,1574-0,1922	0,1845	0,1737-0,1880
	Day 2	Males	0,1528	0,1376-0,1747	0,1533	0,1399-0,1584
		Females	0,1618	0,1471-0,1756	0,1607	0,1432-0,1748
		(Males + Females)	0,1528	0,1465-0,1737	0,1533	0,1416-0,1735
	Day 3	Males	0,1577	0,1422-0,1591	0,1538	0,1480-0,1606
		Females	0,1516	0,1463-0,1728	0,1521	0,1313-0,1644
		(Males + Females)	0,1525	0,1458-0,1635	0,1538	0,1408-0,1634
	Day 4	Males	0,1586	0,1346-0,1599	0,158	0,1560-0,1674
		Females	0,1510	0,1477-0,1604	0,1670	0,1595-0,1754
		(Males + Females)	0,1522	0,1466-0,1592	0,1605	0,1580-0,1735
	Day 5	Males	0,154	0,1456-0,1580	0,1607	0,1502-0,1663
		Females	0,1506	0,1390-0,1758	0,1577	0,1533-0,1656
		(Males + Females)	0,1540	0,1431-0,1575	0,1607	0,1529-0,1663

Table S2. Means and standard deviation of weighs from the different genotypes during different time-points. PND=post-natal day, * or # p<0.05, ** or ## p<0.01 and *** or ### p<0.001. Statistical differences between KO and WT mice are marked with an asterisk (*), between HET and KO mice are marked with a hash (#) sign (repeated measure two-way ANOVA, Bonferroni *post hoc*).

Age	Sex	WT Mean ± SD	HET Mean ± SD	KO Mean ± SD
PND 1	Males	1,452 ± 0,0603		1,423 ± 0,1563
	Females	1,345 ± 0,1043		1,300 ± 0,1533
PND 2	Males	1,668 ± 0,0808		1,669 ± 0,1855
	Females	1,555 ± 0,1212		1,528 ± 0,1574
PND 3	Males	2,058 ± 0,1065		2,040 ± 0,2297
	Females	1,916 ± 0,1744		1,928 ± 0,1879
PND 4	Males	2,539 ± 0,1442		2,421 ± 0,2662
	Females	2,355 ± 0,2126		2,332 ± 0,2617
PND 5	Males	3,007 ± 0,1855		2,868 ± 0,2957

	Females	2,840 ± 0,2736		2,756 ± 0,3319
PND 6	Males	3,487 ± 0,1849		3,268 ± 0,3429
	Females	3,316 ± 0,3273		3,154 ± 0,3787
PND 7	Males	3,960 ± 0,2078		3,558 ± 0,3982
	Females	3,745 ± 0,3267		3,610 ± 0,4445
PND 8	Males	4,372 ± 0,2246		3,756 ± 0,4971
	Females	4,135 ± 0,3717		4,104 ± 0,6561
PND 9	Males	4,648 ± 0,2834		4,063 ± 0,5387
	Females	4,465 ± 0,4282		4,436 ± 0,6933
PND 10	Males	5,004 ± 0,2675		4,460 ± 0,4844
	Females	4,852 ± 0,4391		4,788 ± 0,7375
PND 11	Males	5,343 ± 0,3427		4,784 ± 0,453
	Females	5,203 ± 0,5324		5,184 ± 0,7851
PND 12	Males	5,700 ± 0,3923		5,094 ± 0,4152
	Females	5,588 ± 0,5719		5,534 ± 0,8216
PND 13	Males	6,029 ± 0,4898		5,289 ± 0,4617 *
	Females	5,919 ± 0,6392		5,618 ± 0,863
PND 14	Males	6,277 ± 0,4718		5,530 ± 0,5294 *
	Females	6,101 ± 0,6070		5,796 ± 0,9841
PND 15	Males	6,479 ± 0,4341		5,641 ± 0,5358 **
	Females	6,245 ± 0,5301		5,902 ± 1,105
PND 16	Males	6,613 ± 0,4775		5,690 ± 0,534 **
	Females	6,405 ± 0,6293		6,054 ± 1,073
PND 17	Males	6,734 ± 0,3247		5,863 ± 0,5191 **
	Females	6,440 ± 0,5299		6,152 ± 1,109
PND 18	Males	6,894 ± 0,3780		6,016 ± 0,6189 **
	Females	6,419 ± 0,4477		6,256 ± 1,173
PND 19	Males	7,170 ± 0,3792		6,310 ± 0,6901 **
	Females	6,651 ± 0,5398		6,516 ± 1,140
PND 20	Males	7,526 ± 0,4380		6,680 ±0,7571 **
	Females	6,874 ± 0,5453		6,938 ± 1,226
PND 21	Males	7,704 ± 0,4581		7,148 ± 0,8424
	Females	7,063 ± 0,5798		7,508 ± 1,338
PND 28	Males	12,99 ± 0,9343		10,25 ± 1,513 ***
	Females	11,55 ± 1,0130		10,37 ± 1,610 *
10 weeks	Males	25,85 ± 2,482	25,43 ± 1,187 ##	22,41 ± 2,034 ***
	Females	20,31 ± 1,449	19,55 ± 0,690 #	17,84 ± 1,575 ***
13 weeks	Males	28,32 ± 2,704	27,57 ± 1,072 ##	24,69 ± 1,757 ***
	Females	22,14 ± 1,743	20,74 ± 0,669	19,64 ± 1,525 ***
20 weeks	Males	30,62 ± 2,531	29,59 ± 1,157 ##	26,96 ± 1,977 ***
	Females	24,04 ± 2,047	22,67 ± 1,107 #	20,85 ± 1,696 ***
25 weeks	Males	31,05 ± 2,853	30,24 ± 1,024 #	27,52 ± 1,788 ***
	Females	24,99 ± 2,220	24,48 ± 2,533 #	21,66 ± 1,817 ***

Table S3. Medians and the interquartile range for each parameter in the open field test. Statistical differences between KO and WT mice are indicated with an asterisk sign (*), between HET and WT mice with a section sign (§) and between HET and KO mice with a hash sign (#). p<0.05 = * or § and p<0.01= **, §§ or ## (*post hoc* Mann-Whitney U).

**Parameter**	**Time-point**	**Sex**	**WT** Median	Quartiles	**HET** Median	Quartiles	**KO** Median	Quartiles
Frequency Central circle	0-5	Males	4,00	2,5-8	8,00	2,75-11	6,00	3-11
		Females	4,50	4-7	4,00	3-5,75	8,00	3,5-10,5
		(Males + Females)	4,00	4-7	4,50	3-8,75	6,00	3-9,75
	5-10	Males	6,00	3,5-8,5	11,50	6,75-16,25 §	7,00	3,5-17,5
		Females	7,00	6-8,5	6,50	3-10,75	10,00	8-14,5 *
		(Males + Females)	6,00	4,5-8,5	10,00	4,5-14,75	10,00	5,75-13,75 *
	10-15	Males	5,00	1,5-9	7,00	5-11,5	5,00	2,5-9
		Females	3,50	2-6	7,00	6-9,5 §	8,00	6,5-10,5 **
		(Males + Females)	4,00	2-6,5	7,00	5-10,75 §§	7,00	4,5-9,25 *
	15-20	Males	4,00	3-7,5	5,50	5-10	8,00	3,5-12
		Females	6,00	3-9,5	4,00	2-5,25 #	9,00	4,5-12
		(Males + Females)	6,00	3-8	5,00	3,25-7,75	8,50	4-11,5
	20-25	Males	4,00	2-8	5,00	2-8,75	4,00	3-6
		Females	4,50	2-7,5	4,00	2-8,25	9,00	4,5-10 *
		(Males + Females)	4,00	2-7	4,00	2-7,75	5,50	4-9
	25-30	Males	5,00	2,5-7	6,50	5-8,25	5,00	3-9
		Females	3,00	2-6	4,50	3-11,25	7,00	2,5-11
		(Males + Females)	4,00	2-6	6,00	4-9 §	6,50	3-9
	30-35	Males	3,00	2-7	7,50	4-10 §/#	3,00	0,5-7
		Females	2,50	0,25-3,75	4,00	2,5-7,75	7,00	4,5-10,5 **
		(Males + Females)	3,00	1,5-5,5	5,00	3-8,75 §§	5,00	3-8,5 *
	35-40	Males	2,00	1-3	3,50	2,5-6	3,00	1,5-6
		Females	5,00	1,25-6,75	4,00	2-6,25	7,00	2,5-10
		(Males + Females)	3,00	1-5	4,00	2,25-6	4,50	2-9,25
	40-45	Males	2,00	1-3,5	2,00	1,75-4,25	4,00	1-8
		Females	3,50	2-6,5	4,00	1,75-7	2,00	1,5-5,5
		(Males + Females)	3,00	1,5-4,5	3,00	2-5,75	2,00	1-7,25
	45-50	Males	1,00	0-5	2,50	0-6,25	4,00	2,5-5
		Females	1,50	0-4	4,00	2-9 §	5,00	2,5-7,5 *
		(Males + Females)	1,00	0-4	4,00	1,25-7	4,00	2,75-6,25 *
	50-55	Males	2,00	0-4,5	2,00	1-4,25	2,00	0,5-5,5
		Females	1,00	0-3,75	3,00	1-4,25	2,00	0,5-4
		(Males + Females)	2,00	0-4	3,00	1-4	2,00	0,75-5
	55-60	Males	1,00	0-3,5	2,00	1-4	3,00	1-6
		Females	1,00	0-3	2,50	1,75-6,25	4,00	0-7
		(Males + Females)	1,00	0-3	2,00	1,25-4 §	3,50	0,75-6,25

Duration (s) Central circle	0-5	Males	4,64	2,32-11,8	11,12	4,2-22,5	7,16	2,5-22,26
		Females	6,78	3,58-11,98	5,00	2,39-8,17	16,28	3,84-21,36
		(Males + Females)	4,92	3,4-11,64	7,20	3,15-13,28	9,52	3,09-20,86
	5-10	Males	7,20	4,68-10,4	20,34	5,7-50,43	7,60	3,7-25,94
		Females	7,04	4,44-15,24	8,24	2,41-19,56	23,84	13,82-27,76
		(Males + Females)	7,20	4,68-13,2	13,82	4,37-22,91	17,16	6,9-26,96 *
	10-15	Males	7,52	4,06-19,14	19,02	7,5-34,45	6,96	3,12-34,92
		Females	5,92	2,04-10,5	8,60	4,07-29,33	16,92	8,66-26,02
		(Males + Females)	6,40	3,7-14,92	17,40	5,96-31,26 §	16,86	4,58-27,48
	15-20	Males	8,72	3,54-15,32	23,22	9,3-32,96	22,68	4,54-26,46
		Females	11,90	5,25-17,31	4,58	2,77-11,72	17,56	7,76-40,38
		(Males + Females)	11,56	4,5-17,30	10,96	3,98-26,51	19,22	7,85-30,11
	20-25	Males	7,00	1,5-21,22	14,60	4,97-47,52	9,36	3,52-19,16
		Females	7,48	2,58-13,78	5,34	3,18-12,91	14,60	7,02-30,36
		(Males + Females)	7,08	1,94-13,44	10,34	3,56-19,10	10,86	5,53-26,03
	25-30	Males	5,48	1,28-11,24	8,96	4,23-42,10	9,64	4,2-17,16
		Females	4,44	1,21-11,2	6,08	3,09-21,84	6,56	4,74-35,06
		(Males + Females)	5,12	1,28-11,16	6,64	3,63-30,93 §	8,10	4,91-17,89
	30-35	Males	4,00	1,86-17,74	17,22	4,68-24,01	5,68	0,24-15,42
		Females	4,22	0,19-10,15	7,34	1,68-22,87	8,12	3,54-20,9
		(Males + Females)	4,00	1,26-9,14	10,48	4,2-21,56 §	6,10	3,06-15,17
	35-40	Males	1,44	0,94-5,62	11,90	5,19-23,81	9,52	1,84-14,2
		Females	4,64	0,77-10,20	3,30	2,05-17,97	11,84	3,8-14,42
		(Males + Females)	1,72	0,84-8,48	9,46	2,12-21,9 §	9,58	2,5-13,88 *
	40-45	Males	2,12	1,2-5,38	5,82	1,17-8,44	5,60	3,32-26,84
		Females	3,66	0,97-12,89	5,12	2,26-8,81	1,60	0,88-30,58
		(Males + Females)	2,72	1,2-7,72	5,74	1,9-8,41	4,54	1,47-25,60
	45-50	Males	1,80	0-4,76	4,44	0-9,64	5,44	1,84-12,02
		Females	0,96	0-6,43	5,10	1,8-11,71	5,00	1,72-10,26
		(Males + Females)	1,24	0-5,86	4,74	0,66-10,93 §	5,22	1,86-10,19 *
	50-55	Males	5,28	0-10,24	3,72	1,02-25,59	2,64	0,82-11,26
		Females	0,96	0-2,89	2,96	0,62-4,17	2,08	0,12-5,58
		(Males + Females)	1,96	0-8,76	3,56	0,98-8,97	2,32	0,18-7,96
	55-60	Males	1,24	0-5,24	7,90	1,26-17,07	3,56	0,74-7,38
		Females	1,20	0-3,93	4,50	1,270-15,57	2,32	0-9,72
		(Males + Females)	1,24	0-4,9	4,98	1,29-14,66 §§	3,02	0,36-8,11
Duration per visit (D/F; s) Central circle	0-5	Males	1,23	0,824-2,077	1,23	0,89-3,25	1,39	0,89-1,89
		Females	1,33	0,84-1,771	1,10	0,88-1,43	1,64	0,99-2,15
		(Males + Females)	1,28	0,85-1,92	1,15	0,89-1,83	1,54	0,94-1,99
	5-10	Males	1,21	0,84-2,11	1,81	0,89-2,86	1,09	0,88-1,35
		Females	1,17	0,76-2,06	0,96	0,67-1,73	1,83	1,35-2,65
		(Males + Females)	1,21	0,84-2,06	1,30	0,88-2,33	1,35	1,05-2,05
	10-15	Males	1,62	0,89-3,2	2,61	1,58-3,92	1,84	1,03-4,92
		Females	1,29	0,74-3,18	1,32	0,92-2,53	2,35	1,08-2,44
		(Males + Females)	1,37	0,78-3,11	1,84	1,17-3,71	2,02	1,09-2,96

	15-20	Males	2,05	0,55-3,04	3,09	1,99-5,18	2,01	1,14-2,79
		Females	1,71	1,28-2,73	1,48	0,77-2,6	2,06	1,3-3,83
		(Males + Females)	1,85	1,04-2,92	2,24	1,08-3,22	2,04	1,32-3,17
	20-25	Males	1,65	0,76-2,72	4,32	1,84-5,7	2,34	1,12-3,68
		Females	1,09	0,74-4,62	1,19	0,91-2,37	1,71	1,01-3,04
		(Males + Females)	1,18	0,76-2,79	2,28	1,11-4,41	1,86	1,01-3,07
	25-30	Males	1,34	0,52-1,69	1,63	0,84-5,5	1,61	0,91-1,98
		Females	1,23	0,42-2,02	1,26	0,95-1,96	1,33	0,77-3,11
		(Males + Females)	1,25	0,52-1,71	1,31	0,86-2,12	1,47	0,8-2,4
	30-35	Males	1,00	0,69-2,88	2,15	0,99-3,05	1,30	0,24-1,91
		Females	1,25	0,19-2,27	1,60	1,06-2,47	1,62	0,76-1,98
		(Males + Females)	1,00	0,67-2,34	1,73	1-2,54	1,47	0,49-1,9
	35-40	Males	1,00	0,4-1,5	3,04	1,63-5,5	1,82	1,26-3,21
		Females	1,06	0,42-1,92	1,29	0,57-2,34	1,28	0,96-1,91
		(Males + Females)	1,00	0,41-1,5	2,13	0,87-3,82 §§	1,54	1,15-2,24 *
	40-45	Males	0,84	0,56-1,83	2,00	1-3,1	2,44	1,09-4,19
		Females	1,06	0,33-2,0	1,43	0,85-2,81	1,01	0,35-3,35
		(Males + Females)	0,91	0,53-1,87	1,58	0,92-2,97 §	1,50	0,6-4,19
	45-50	Males	0,79	0-1,05	1,09	0-2,5	1,52	0,59-2,05
		Females	0,52	0-1,48	0,94	0,53-1,63	1,00	0,69-1,46
		(Males + Females)	0,69	0-1,15	0,98	0,5-1,72	1,18	0,67-1,7 *
	50-55	Males	1,76	0-2,44	1,66	0,95-4,7	1,08	0,43-3,24
		Females	0,57	0-1,33	0,71	0,4-1,31	0,66	0,09-1,69
		(Males + Females)	0,68	0-1,92	1,04	0,57-2,26	0,87	0,14-2,36
	55-60	Males	0,31	0-2,25	2,49	0,52-6,92	0,83	0,73-1,34
		Females	0,63	0-1,92	1,39	1,1-2,49	0,48	0-1,84
		(Males + Females)	0,36	0-1,93	1,57	0,81-3,54 §§	0,81	0,29-1,33 ##
Distance moved (cm) Central circle	0-5	Males	43,08	24,93-118,3	109,20	47,38-148,7	89,39	35,65-142
		Females	68,42	47,54-101,9	60,53	31,52-87,22	91,43	47,22-148,1
		(Males + Females)	56,48	39,72-101,3	77,41	43,63-133,2	90,41	35,92-143,7
	5-10	Males	57,20	41,31-105,2	148,40	77,37-192,4 §	90,13	54,98-276,5
		Females	85,71	50,58-137	75,96	45,05-154,8	132,00	117,2-217,3 *
		(Males + Females)	73,25	44,47-112,8	122,30	60,96-168	124,90	71,13-235,2 *
	10-15	Males	58,49	32,21-117,7	106,00	79,57-211,5 §	62,07	20,8-147,2
		Females	67,62	24,37-88,68	100,60	68,97-120,2 §	116,20	79,3-164,8 **
		(Males + Females)	62,81	27,81-86,45	100,60	79,16-154,3 §§	111,60	47,19-161,3
	15-20	Males	63,01	48,66-73,62	97,73	67,69-161,9 §	94,05	46,79-163,5
		Females	83,03	52,15-140,4	52,04	30,2-100,6 #	90,47	61,84-189,2
		(Males + Females)	66,46	49,99-92,63	78,53	46,33-133,5	92,26	62,18-166,7
	20-25	Males	58,36	18,51-111,7	73,60	40,60-131,4	61,74	36,44-94,06
		Females	67,38	26,06-83,43	50,69	24,92-100,1	100,40	56,47-181,5
		(Males + Females)	63,66	23,59-82,88	64,82	36,23-106	73,34	54,98-120,7
	25-30	Males	56,93	26,93-81,52	68,62	49,34-137,8	52,88	43,21-114,1
		Females	50,24	26,37-101	54,80	35,9-92,08	95,45	42,43-181,8
		(Males + Females)	53,24	28,66-98,11	63,83	46,94-110,1	89,18	44,16-131
	30-35	Males	46,28	23,37-95,21	86,86	69,37-106,3 #	52,35	5,59-67,88

		Females	36,60	3,83-73,56	59,19	35,8-106,1	77,03	58,55-148,6 [**]
		(Males + Females)	39,27	17-78,68	74,18	47,82-97,92 [§]	67,88	41,07-93,35
	35-40	Males	17,08	9,29-54,25	56,12	26,7-91,81 [§]	51,69	22,54-72,13
		Females	55,40	19,15-97,42	39,98	29,82-90,61	89,39	41,9-129,9
		(Males + Females)	33,43	12,52-78,75	44,84	28,34-89,68	60,12	28,06-107,5
	40-45	Males	28,99	16,92-41,19	35,90	19,43-53,76	52,04	21,34-103,5
		Females	42,96	17,49-79,52	54,23	25,12-96,67	19,89	12,53-90,05
		(Males + Females)	38,46	16,92-58,1	37,72	21,88-86,82	43,10	18,21-102,4
	45-50	Males	17,99	0-73,76	33,04	0-60,33	62,89	24,30-78,6
		Females	15,97	0-47,38	67,11	19,05-110,7 [§]	82,41	37,13-104,7 [*]
		(Males + Females)	17,99	0-56,48	40,86	15,13-80,41	63,17	30,37-98,41 [*]
	50-55	Males	45,17	0-84,83	22,12	16,52-71,17	36,45	8,64-67,07
		Females	15,35	0-46,28	37,47	16,98-55,95	28,12	0,18-48,17
		(Males + Females)	22,36	0-49,49	30,33	17,54-58,52	30,51	0,27-56,49
	55-60	Males	18,72	0-39	37,45	24,24-64,22	30,11	15,48-67,71
		Females	13,17	0-46,09	40,17	20,87-66,41 [§]	63,85	0-94,23
		(Males + Females)	16,26	0-43,09	38,81	24,06-62,19 [§§]	34,50	9,71-74,67
Mean velocity (cm/s) Central circle	0-5	Males	8,76	6,35-17,43	6,51	3,01-11,38	11,89	8,48-14,55
		Females	12,55	9,57-18,7	10,52	7,77-11,7	8,61	5,92-13,26
		(Males + Females)	12,17	7,37-17,68	9,16	5,55-11,46	10,84	6,73-14,3
	5-10	Males	14,66	6,26-19,51	8,21	5,2-12,57	11,39	6,4-13,4
		Females	9,20	5,11-16,88	10,51	7,05-17,16	7,41	4,46-9,12
		(Males + Females)	10,52	5,7-18,23	8,98	6,68-14,36	8,71	6,11-12,21
	10-15	Males	9,56	7,05-22,45	6,33	4,18-10,44	7,25	3,51-10,26
		Females	13,13	6,19-18,18	8,92	4,5-14,61	8,77	3,54-11,24
		(Males + Females)	11,44	7,56-16,24	6,48	4,19-12,19	8,15	3,55-10,5
	15-20	Males	11,73	7,26-25,37	4,78	3,17-8,63	6,27	4,61-8,19
		Females	7,72	6,3-11,23	7,60	5,4-18	5,95	4,26-10,95
		(Males + Females)	9,77	6,73-12,5	5,85	4,78-11,42	6,04	4,91-8,19
	20-25	Males	6,69	4,55-16,81	3,40	2,39-5,05	7,34	4,52-9,35
		Females	9,09	6,06-14,52	8,06	6,38-12,67	6,64	6,27-9,8
		(Males + Females)	7,21	5,04-13,19	5,89	3,4-8,2	6,74	5,91-9,35
	25-30	Males	9,46	5,27-10,44	3,57	2,53-9,37	8,65	6,67-9,82
		Females	8,31	7,58-16,69	7,34	5,55-12,86	10,00	4,47-16,07
		(Males + Females)	8,88	6,93-11,41	5,91	3,57-11,03	8,65	5,69-11,53
	30-35	Males	9,98	3,29-11,47	4,71	4,24-5,8	7,43	3,21-12,44
		Females	9,36	5,79-18,8	6,98	3,69-11,17	8,93	3,66-9,55
		(Males + Females)	9,67	4,6-13,28	5,73	4,16-8,03	8,80	3,21-10,01
	35-40	Males	17,03	7,92-25,62	3,59	2,04-9,2	6,85	4,12-12,15
		Females	8,36	5,26-12,87	7,47	4,99-18,81	7,20	5,89-13,97
		(Males + Females)	10,08	7,87-18,36	6,94	3,17-13,38	7,20	5,15-12,15
	40-45	Males	10,71	6,58-15,02	6,88	4,8-9,74	8,29	3,74-10,86
		Females	12,05	4,52-14,74	11,14	5,19-14,76	12,57	5,42-24,85
		(Males + Females)	11,38	5,05-14,91	8,93	5,54-11,54	8,83	3,88-13,43
	45-50	Males	15,43	10,82-19,75	7,18	4,61-11,48	9,60	5,79-13,2

		Females	12,76	8,02-18,11	10,76	6,35-16,56	10,21	7,33-18,14
		(Males + Females)	14,09	8,8-17,68	9,09	6,13-14,23	10,05	6,39-13,2
	50-55	Males	5,38	4,91-9,88	5,75	3,56-15,3	8,01	3,91-15,9
		Females	22,09	8,42-39,32	13,94	9,09-17,84	17,60	8,76-24,49
		(Males + Females)	9,02	5,27-22,46	12,48	5,32-16,97	12,52	5,3-19,39
	55-60	Males	13,40	4,43-27,54	9,50	3,01-10,1	13,27	6,98-18,92
		Females	8,30	4,17-18,23	8,97	2,7-11,58	11,93	4,57-27,43
		(Males + Females)	10,85	4,61-21,66	9,43	2,78-10,73	12,36	6,98-18,92
Duration (s) Wall zone	0-5	Males	265,80	241,1-282,4	238,30	215,8-258,1	258,00	217,6-273,2
		Females	264,00	255,6-278,1	273,10	243,2-288,8	254,00	216,4-279,3
		(Males + Females)	265,80	250,9-280,3	251,30	224-278,4	256,10	223,3-276,8
	5-10	Males	255,90	238,3-263,1	209,50	171,8-222,2	229,90	142,8-255,4
		Females	248,50	230,7-273,4	257,30	219-266,8	199,60	185,2-239,8
		(Males + Females)	254,60	232,4-269,1	220,90	187,5-258,4 §	205,90	170,5-250,8 **
	10-15	Males	251,40	217,6-270,2	213,10	171,2-233,2	206,10	190,5-266,7
		Females	251,30	229,1-271	255,30	184,6-269,2	215,80	180,3-241,1
		(Males + Females)	251,40	229,2-270,3	222,40	181,1-264,2	211,00	186,1-249,3 *
	15-20	Males	260,30	222,8-282,9	222,40	176,6-239,8	198,80	183,8-264,3
		Females	247,80	232,5-262,3	263,70	240,4-275,6	224,00	171,2-244,1
		(Males + Females)	256,10	226,9-267,1	241,20	211,2-264,4	211,40	179,5-250,6 *
	20-25	Males	264,70	210,2-273,4	237,40	179,1-257,1	243,80	225,8-255,9
		Females	251,90	229,8-276,3	252,60	222,2-269,8	245,60	203,3-254,6
		(Males + Females)	262,10	221,4-273,4	242,40	201,8-260,1	244,70	220,8-253,3
	25-30	Males	263,50	219,4-280,3	209,70	149,8-251,6	205,80	165,3-263,6
		Females	267,50	235,1-285	246,20	194,2-267,7	239,70	181,5-256,3
		(Males + Females)	266,60	236,6-283,3	234,20	173,9-264,5 §§	230,20	166,2-255,3 **
	30-35	Males	241,10	163,9-288,1	204,90	181,7-247,5	251,90	191,3-290,3
		Females	282,90	238,6-290,3	253,70	194,8-271,9	230,50	205,6-276,1
		(Males + Females)	256,20	229,9-290,3	231,00	187,8-262 §	246,20	206,2-284,7
	35-40	Males	277,70	252,6-293,2	246,50	211,5-264,5	234,20	195,1-284,9
		Females	241,70	205,1-271,7	271,40	215,8-277,7	244,00	220,7-171,9
		(Males + Females)	263,10	224,7-287,1	258,50	216,3-272,7	243,80	212,6-272,7
	40-45	Males	277,50	254,4-287,3	260,10	215,8-285	259,60	187,1-280
		Females	268,20	255-289,1	267,80	241,7-278,2	260,30	203,4-290,7
		(Males + Females)	271,20	256-287,6	266,80	230-283,1	260,00	195,5-290,4
	45-50	Males	280,00	242,7-297,6	237,30	202,6-282,6	225,30	181,5-258,6
		Females	281,20	248,4-296,9	252,50	228,5-271,6	243,30	200,5-265,8
		(Males + Females)	280,00	246,9-296,4	246,50	212,4-276,4 §§	242,20	185,7-265,3 **
	50-55	Males	279,30	251,1-297,3	229,80	200-274,5	264,60	191,7-291,1
		Females	281,10	252,9-296,4	259,60	237,5-279,7	249,00	243,6-286,9
		(Males + Females)	279,30	253,1-297,1	251,70	221,1-276,6 §	252,10	239,5-285,1
	55-60	Males	280,00	262,1-294,6	253,40	201,5-286,4	190,60	132,6-287,5
		Females	290,60	268,3-296,7	261,80	235-292,3	277,90	205,4-291,9
		(Males + Females)	283,60	264,9-295,9	261,80	229,1-289,5 §§	237,90	168,5-291,1 *
Total distance	0-5	Males	1409,00	1145-1848	1610,00	1227-1965	1454,00	1310-2056
		Females	1766,00	1402-1854	1597,00	1377-1904	1859,00	1310-2204

(cm) Arena	5-10	Males	1451,00	1220-1729	1518,00	1334-1907	1577,00	1200-1739
		Females	1500,00	1365-1824	1620,00	1497-1953	1799,00	1436-2075
	10-15	Males	1450,00	1284-1742	1562,00	1190-1696	1432,00	1078-1712
		Females	1611,00	1382-1704	1768,00	1363-1925	1613,00	1283-1889
	15-20	Males	1431,00	977,1-1535	1334,00	1130-1624	1384,00	713,3-1731
		Females	1465,00	1289-1874	1399,00	1202-1625	1578,00	1320-1947
	20-25	Males	1301,00	1007-1674	1214,00	894,7-1335	1138,00	926,9-1256
		Females	1380,00	1257-1608	1423,00	1132-1802	1445,00	1231-1792
	25-30	Males	1395,00	689,2-1557	1169,00	1004-1322	1289,00	845,2-1373
		Females	1420,00	1056-1699	1478,00	1124-1649	1427,00	1044-1707
	30-35	Males	1212,00	843,8-1484	1134,00	976,2-1271	1085,00	506,4-1139
		Females	1421,00	945,3-1567	1142,00	883,2-1501	1247,00	1033-1934
	35-40	Males	860,50	706,1-1441	992,70	662,7-1132	985,70	710,1-1096
		Females	1048,00	751-1430	1240,00	988-1516	1144,00	993,4-1468
	40-45	Males	904,40	594,3-1022	993,60	723,4-1075	1031,00	423,6-1123
		Females	1025,00	818,6-1424	1194,00	904,7-1548	1162,00	757,5-1532
	45-50	Males	1058,00	340,6-1373	868,50	701,5-1033	1018,00	703,2-1232
		Females	844,00	569,6-1055	1219,00	1032-1484	1075,00	738,9-1452
	50-55	Males	676,20	434,4-1209	859,60	697,7-1268	765,60	550,8-1012
		Females	940,00	445,6-1181	1125,00	830,1-1421	956,70	632-1148
	55-60	Males	772,70	589,6-1031	737,10	594-888,2	836,90	538,1-1070
		Females	723,80	410,3-1043	853,10	700,9-1249	1011,00	459,5-1348
Rearing	0-5	Males	23,00	16,5-31,5	20,00	17-29	20,00	16-31,25
		Females	27,00	20-33	23,00	19-27	23,00	13,75-37
		(Males + Females)	24,00	20-33	22,50	17,75-29	21,50	15,25-32
Grooming	0-5	Males	2,00	1-3,5	2,00	1-2	1,50	1-2
		Females	2,00	1-3	2,00	2-3	1,00	1-2,25
		(Males + Females)	2,00	1-3	2,00	1-3	1,00	1-2

Table S4. Calculated effect sizes for significant results in the open field test for parameters with interaction effect of Sex:Genotype:Time, ranging from small (0.1-0.3), intermediate (0.3-0.5) to large (>0.5) effects (n=13 WT males, 12 WT females, 14 HET males, 14 HET females, 9 KO males and 9 KO females).

Parameter	Time-point (min)	Comparison	P-value	Effect size
Central circle distance	15-20	m-HET vs f-HET	<0.05	0,39
	30-35	m-KO vs f-KO	<0.05	-0,52
	5-10	m-WT vs m-HET	<0.05	-0,45
	10-15	m-WT vs m-HET	<0.05	-0,42
	15-20	m-WT vs m-HET	<0.05	-0,46
	35-40	m-WT vs m-HET	<0.05	-0,41
	30-35	m-HET vs m-KO	<0.01	0,58
	10-15	f-WT vs f-HET	<0.05	-0,44
	45-50	f-WT vs f-HET	<0.05	-0,43
	55-60	f-WT vs f-HET	<0.05	-0,42
	5-10	f-WT vs f-KO	<0.05	-0,50
	10-15	f-WT vs f-KO	<0.01	-0,55
	30-35	f-WT vs f-KO	<0.01	-0,55
	45-50	f-WT vs f-KO	<0.05	-0,51
	15-20	f-HET vs f-KO	<0.05	-0,41
Central circle frequency	15-20	m-HET vs f-HET	<0.05	0,39
	20-25	m-KO vs f-KO	<0.05	-0,49
	5-10	m-WT vs m-HET	<0.05	-0,48
	30-35	m-WT vs m-HET	<0.05	-0,45
	30-35	m-HET vs m-KO	<0.05	0,41
	10-15	f-WT vs f-HET	<0.05	-0,47
	45-50	f-WT vs f-HET	<0.05	-0,46
	5-10	f-WT vs f-KO	<0.05	-0,54
	10-15	f-WT vs f-KO	<0.01	-0,58
	20-25	f-WT vs f-KO	<0.05	-0,47
	30-35	f-WT vs f-KO	<0.01	-0,66
	45-50	f-WT vs f-KO	<0.05	-0,45
	15-20	f-HET vs f-KO	<0.05	-0,46

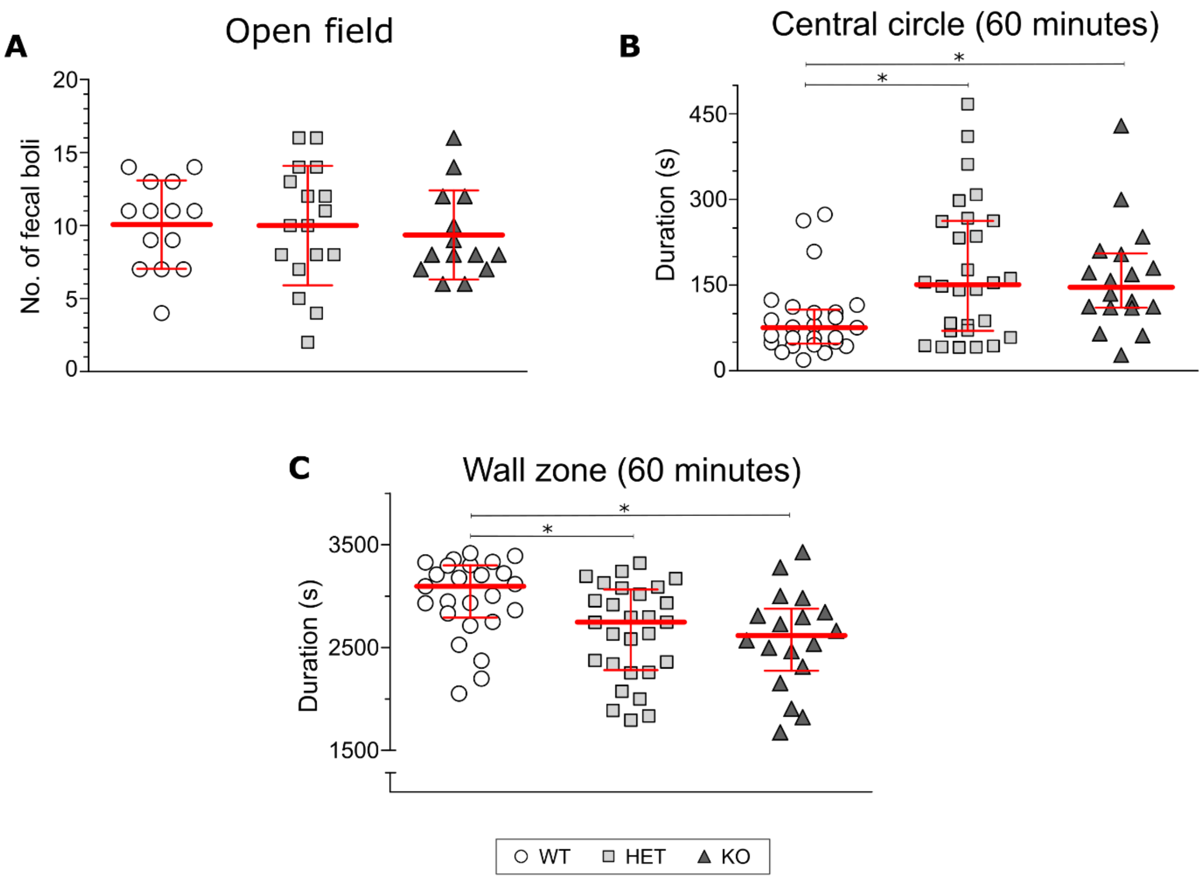

Figure S1. Cumulative data from 60 minutes in open field test by mice deficient in the SLC38A10 protein and WT controls. (A) Number of fecal boli after 60 minutes in the open field test. Data are illustrated as mean values ± standard deviation for male (7 WT, 11 HET, 8 KO) and female mice (7 WT, 6 HET, 6 KO) together. Statistical analysis was made with one-way ANOVA, but no statistical differences were found (WT: 10.07 ± 3.025, HET: 10.00 ± 4.093 and KO: 9.357 ± 3.054). **(B)** Cumulative duration in the central circle of the open field arena (WT: 75.6, IQR 47.5-107.3; HET: 151.2, IQR 70.22-262.5; KO: 146.4, IQR 110.5-205.5) and **(C)** cumulative duration in the wall zone of the open field arena (WT: 3096, IQR 2791-3300; HET: 2747, IQR 2280-3065; KO: 2618, IQR 2275-2878) are illustrated for both sexes and as medians with interquartile range (males: 13 WT, 14 HET, 9 KO; females: 12 WT, 14 HET, 9 KO). Analyses were made with Kruskal-Wallis, *post-hoc* Dunn's multiple comparison, $*p<0.05$.

Table S5. Medians and the interquartile range for each parameter in the elevated plus maze. Statistical differences between WT and Het mice are indicated with a section (§) sign, § $p<0.05$ (Kruskal-Wallis, Dunn's multiple comparison *post hoc*).

**Parameter**	**Sex**	**WT** Median	Quartiles	**HET** Median	Quartiles	**KO** Median	Quartiles
Frequency open arms	Males	2.5	0.0-10.25	3.0	1.0-8.0	2.5	0.0-6.75
	Females	1.5	0.75-3.25	4.0	3.0-8.0	5.0	0.75-5.0
	(Males + Females)	1.5	0.0-5.75	4.0	1.0-8.0	4.0	0.0-5.25
Duration (s) open arms	Males	1.64	0.0-90.15	15.76	3.72-67.72	2.18	0.0-45.61
	Females	9.86	1.44-19.24	16.68	3.08-35.84	36.34	5.49-56.61
	(Males + Females)	3.58	0.0-38.58	16.22	3.56-46.97	14.06	0.0-51.06
Frequency closed arms	Males	28.5	21.75-34.75	34.0	26.0-42.0	29.0	24.75-38.5
	Females	26.0	22.25-34.25	33.0	30.0-40.0	29.5	25.25-30.75
	(Males + Females)	28.0	22.25-34.25	33.5	28.75-40.0 §	29.0	25.75-32.25
Duration (s) closed arms	Males	468.0	382.2-530.8	464.1	408.2-509.5	449.8	412.9-490.0
	Females	470.7	433.2-501.4	468.3	449.3-506.6	468.6	438.5-494.9
	(Males + Females)	470.7	384.0-505.4	464.3	445.0-507.0	459.0	429.8-490.0
Frequency (sum of all zones)	Males	65.0	47.75-76.75	77.0	53.0-91.0	59.0	56.0-79.25
	Females	53.5	46.5-75.0	75.0	65.0-95.0	63.5	58.75-77.25
	(Males + Females)	58.5	47.25-75.0	75.0	63.0-92.0 §	61.0	56.75-74.0
Frequency center	Males	32.0	23.5-37.5	38.0	26.0-46.0	29.5	28.0-39.5
	Females	26.5	23.25-37.0	37.0	32.0-47.0	32.0	29.25-38.5
	(Males + Females)	29.0	23.5-37.0	37.0	31.75-46.25 §	30.5	28.0-37.0
Duration center (s)	Males	116.2	69.23-145.6	99.68	86.76-135.9	126.4	110.0-159.6
	Females	123.8	91.72-154.4	103.0	91.4-111.2	97.12	86.07-116.8
	(Males + Females)	121.7	83.14-143.2	101.3	89.1-129.1	115.5	91.38-141.4
Duration per visit (s) open arms	Males	0.33	0.0-9.01	5.38	3.72-8.47	0.8	0.0-4.9
	Females	3.24	1.44-9.58	3.73	0.62-6.68	7.89	1.52-10.3
	(Males + Females)	1.93	0.0-8.53	4.33	1.24-7.28	3.06	0.0-8.45
Duration (%) open arms	Males	0.28	0.0-15.03	2.63	0.62-11.29	0.37	0.0-7.6
	Females	1.65	0.24-3.21	2.78	0.51-5.97	6.06	0.92-9.44
	(Males + Females)	0.6	0.0-6.43	2.71	0.59-7.83	2.35	0.0-8.51
Duration (%) closed arms	Males	78.0	63.7-88.46	77.35	68.03-84.92	74.97	68.82-81.67
	Females	78.45	72.2-83.57	78.05	74.89-84.43	78.09	73.08-82.48
	(Males + Females)	78.45	63.99-84.24	77.38	74.17-84.5	76.5	71.63-81.67

Table S6. Medians and the interquartile range for each parameter in the rotarod, challenging beam walk, Y-maze, marble burying and grip strength tests. No statistical differences were found (Kruskal-Wallis, Dunn's multiple comparison *post hoc*).

Test	Sex	WT		HET		KO	
		Median	Quartiles	Median	Quartiles	Median	Quartiles
Rotarod	Males	1,553	0,958-2,693	2,552	1,051-3,142	1,105	0,733-3,16
	Females	2,362	1,777-3,392	2,965	1,946-4,447	2,099	0,762-4,329
	(Males + Females)	2,116	1,396-2,997	2,775	1,645-3,524	1,379	0,759-3,897
Challenging beam walk	Males	0,191	0,167-0,333	0,273	0,222-0,350	0,280	0,150-0,370
	Females	0,257	0,189-0,308	0,211	0,188-0,227	0,143	0,087-0,238
	(Males + Females)	0,250	0,174-0,317	0,222	0,198-0,273	0,221	0,090-0,303
Y-maze Alternation (%)	Males	59,820	50,36-69,65	69,570	65,33-75	60,000	54,96-71,69
	Females	63,160	58,47-74,51	69,570	57,52-73,04	68,480	61,19-70,36
	(Males + Females)	62,500	55,56-71,88	69,570	62,7-73,04	64,410	56,08-70,44
Y-maze No. of entries	Males	25,50	21,75-29,00	25,00	23,50-33,00	30,50	22,00-36,00
	Females	33,00	24,50-38,50	33,00	27,00-39,00	33,00	29,00-38,25
	(Males + Females)	27,00	23,00-34,00	30,00	25,00-34,00	32,00	27,25-36,75
Marble burying	Males	1,50	0,00-4,25	3,00	0-10,25	0,00	0,00-4,00
	Females	0,00	0,00-4,00	0,00	0,00-2,00	1,00	0,00-4,00
	(Males + Females)	0,50	0,00-4,00	0,00	0,00-5,50	0,00	0,00-4,00
Grip strength	Males	6,820	4,085-7,790			8,410	5,130-8,940
	Females	9,230	8,970-9,730			10,440	8,478-11,560
	(Males + Females)	8,490	6,795-9,230			8,650	5,350-10,400
